# Adaptations in energy metabolism and gene family expansions revealed by comparative transcriptomics of three Chagas disease triatomine vectors

**DOI:** 10.1186/s12864-018-4696-8

**Published:** 2018-04-27

**Authors:** Jesús Martínez-Barnetche, Andrés Lavore, Melina Beliera, Juan Téllez-Sosa, Federico A. Zumaya-Estrada, Victorio Palacio, Ernestina Godoy-Lozano, Rolando Rivera-Pomar, Mario Henry Rodríguez

**Affiliations:** 1 Centro de Investigación Sobre Enfermedades Infecciosas, Instituto Nacional de Salud Pública, Cuernavaca, México; 2 Centro de Bioinvestigaciones (CeBio) and Centro de Investigación y Transferencia del Noroeste de Buenos Aires (CITNOBA-CONICET), Universidad Nacional del Noroeste de la Provincia de Buenos Aires, Pergamino, Argentina; 3 Laboratorio de Genética y Genómica Funcional. Centro Regional de Estudios Genómicos. Facultad de Ciencias Exactas, Universidad Nacional de La Plata, La Plata, Argentina

**Keywords:** Chagas disease, Reduviid bugs, Transcriptome, metabolism, oxidative phosphorylation

## Abstract

**Background:**

Chagas disease is a parasitic infection caused by *Trypanosoma cruzi*. It is an important public health problem affecting around seven to eight million people in the Americas. A large number of hematophagous triatomine insect species, occupying diverse natural and human-modified ecological niches transmit this disease. Triatomines are long-living hemipterans that have evolved to explode different habitats to associate with their vertebrate hosts. Understanding the molecular basis of the extreme physiological conditions including starvation tolerance and longevity could provide insights for developing novel control strategies. We describe the normalized cDNA, full body transcriptome analysis of three main vectors in North, Central and South America, *Triatoma pallidipennis*, *T. dimidiata* and *T. infestans*.

**Results:**

Two-thirds of the de novo assembled transcriptomes map to the *Rhodnius prolixus* genome and proteome. A *Triatoma* expansion of the calycin family and two types of protease inhibitors, pacifastins and cystatins were identified. A high number of transcriptionally active class I transposable elements was documented in *T. infestans*, compared with *T. dimidiata* and *T. pallidipennis*. Sequence identity in *Triatoma-R. prolixus* 1:1 orthologs revealed high sequence divergence in four enzymes participating in gluconeogenesis, glycogen synthesis and the pentose phosphate pathway, indicating high evolutionary rates of these genes. Also, molecular evidence suggesting positive selection was found for several genes of the oxidative phosphorylation I, III and V complexes.

**Conclusions:**

Protease inhibitors and calycin-coding gene expansions provide insights into rapidly evolving processes of protease regulation and haematophagy. Higher evolutionary rates in enzymes that exert metabolic flux control towards anabolism and evidence for positive selection in oxidative phosphorylation complexes might represent genetic adaptations, possibly related to prolonged starvation, oxidative stress tolerance, longevity, and hematophagy and flight reduction. Overall, this work generated novel hypothesis related to biological adaptations to extreme physiological conditions and diverse ecological niches that sustain Chagas disease transmission.

**Electronic supplementary material:**

The online version of this article (10.1186/s12864-018-4696-8) contains supplementary material, which is available to authorized users.

## Background

Chagas disease is an important neglected tropical disease in Central and South America. The causative agent is the protozoan *Trypanosoma cruzi*, transmitted to humans by hematophagous insects of the Reduviidae family. Among the Reduviidae, the Triatominae subfamily conforms one of 21 subfamilies. Triatomines are obligatory blood feeders evolved from predatory ancestors. The Rhodniini and Triatomini tribes contain most species within the subfamily. The Rhodniini tribe is an accepted monophyletic group [1], while paraphyly of the Triatomini tribe is in debate [2, 3].

With few exceptions, triatomine species occurs in the Americas occupying a large variety of ecological niches. In this complex mosaic of natural and human-modified ecotopes, triatomines have evolved to explode different habitats associated with their vertebrate hosts. *Rhodnius* species mostly dwell in palm tree canopies were they feed on birds, while *Triatoma* species are land dwellers, living in crevices and burrows were they feed on birds and mammals [4]. Most triatomines are sylvatic, but they have also adapted to domestic and peridomestic habitats, in close relationship with humans. This extended distribution, their longevity and the ability for vectoring *T. cruzi* result in Chagas disease endemism in Latin America, spanning from Northern México to Southern Argentina with an estimated seven to eight million people infected [5].

Four species are relevant vectors of Chagas disease in the Americas: *Rhodnius prolixus, Triatoma dimidiata*, *T. pallidipennis*, and *T. infestans*. *R. prolixus,* a model organism for insect physiology, is a main vector in Venezuela, Colombia, Peru and whole Central America [6, 7]. *Triatoma dimidiata*, consist of several variants occupying sylvatic, peri-domestic and domestic habitats, making a main Chagas disease vector in Central America and Mexico [8–10]*. Triatoma pallidipennis* stands out by showing the capacity to colonize domestic environments in at least 11 Mexican States with a *T. cruzi* infection prevalence up to 90% [11, 12]. The most widespread Chagas disease vector in South America is *T. infestans*, largely adapted to domiciliary habitats, but with sylvatic populations that repopulate the human environment. This is one of the reasons for failure of the indoor insecticide spraying strategy deployed in South America endemic countries [13].

New strategies for Chagas control are urgently needed; information on the genetic basis of their biological adaptations to extreme physiological conditions such as prolonged starvation periods, their extended life span and their capability to adapt to diverse ecological niches could provide insights for novel control interventions. However, despite of their medical significance, detailed genetic information in triatomines is still scarce. The genome of *R. prolixus* has been sequenced [14], but genomic information of the relevant vectors of the *Triatoma* genus is still limited to organ-specific transcriptomes or selected gene families [15–21]. Thus, we still lack information about the gene composition in triatomines to compare protein families and biological processes, and, therefore, a comprehensive picture of the vectors physiology and evolution.

Transcriptome analysis of organisms without sequenced genomes provides a preliminary catalogue, useful for gene discovery, including specific molecules and their putative functions for the comparative analysis among related organisms, as well as a means for gene prediction validation in future genome projects. We generated transcriptomes derived from normalized *T. infestans, T. dimidiata* and *T. pallidipennis* cDNA libraries from all stages of their life cycle and compared them with the genome of *R. prolixus.* This allowed the identification of a large set of shared genes, gene expansions and higher sequence divergence in energy metabolism-related genes, possibly related to adaptations to their life styles, providing the basis for a better understanding of triatomine biology and insights for development of novel control strategies.

## Results

### Sequencing metrics and assembly

Totals of 164.6, 112.8 and 202.6 megabases of raw sequence data were generated for *T. infestans, T. dimidiata* and *T. pallidipennis,* respectively, which resulted in a cDNA assembly of 3904, 4847 and 5148 isogroups (unigenes) containing from 35% to 69% of assembled reads using the Newbler assembler (Table 1). Most isogroups contained a single isotig (transcript isoform). Mean isotig length was 841, 840 and 893 bp in comparison to 1017 bp in *R. prolixus*, with a corresponding N50 of 871, 880 and 921 bp, respectively (Table 1). Given that we used the long read GS FLX+ 454-Roche system, singletons that mapped with the *R. prolixus* genome or proteome were also included to improve gene discovery. The final dataset included 35,629, 29,024 and 31,175 transcripts for *T. infestans, T. dimidiata* and *T. pallidipennis*, respectively. The G + C % in the *Triatoma* transcriptomes (Table 1) was slightly lower than in *R. prolixus* transcript dataset (39.84 ± 6.79%)*.*

**Table 1:** Sequencing metrics

	*T. dimidiata*		*T. infestans*		*T. pallidipennis*	
Raw sequencing						
Raw reads	358,962		559,962		626,401	
Filtered reads (PRINTSEQ)	237,226	66.1%	347,620	62.1%	421,667	67.3%
Number of bases (Mbps)	112.8		164.6		202.6	
Assembly						
Aligned reads	173,525	73.3%	161,674	46.6%	353,083	83.8%
Aligned bases (Mbps)	82.3	73.1%	74.2	45.1%	167.2	82.6%
Assembled reads	140,289	59.3%	124,306	35.8%	291,051	69.1%
Partially assembled reads	33,138	14.0%	37,334	10.8%	61,847	14.7%
Singletons	56,397	23.8%	174,467	50.3%	54,964	13.1%
Isogroups	4847		3904		5148	
Average contig count	1.6		1.7		1.8	
Average isotig count	1.3		1.2		1.3	
Largest isotig count	15		30		18	
Number of isotigs	6212		4856		6476	
Average contig count	1.6		1.5		1.5	
Largest contig count	7		9		9	
Isotigs with one contig	3824		3232		4169	
Number of bases (Mbps)	5.2		4.1		5.8	
Average isotig size	840		841		893	
N50 isotig size	880		871		921	
Largest isotig size	6814		3682		4465	
Q40 plus bases (Mbps)	3.5	95.8%	2.9	95.9%	4.0	95.4%
< Q39 bases (Mbps)	0.2	4.2%	0.1	4.1%	0.2	4.6%
*full_dataset*						
Total sequences	29,024		35,629		31,175	
Contigs/isotigs	6217	21%	4886	14%	6530	21%
Singletons	22,807	79%	30,743	86%	24,645	79%
Total bases (Mbps):	16.9		20.9		18.2	
% G + C	33.73	± 5.48%	35	± 6.12%	33.7	± 5.75%
*nr_dataset* (1 isotig per isogroup)						
Total sequences	27,652		34,646		29,789	
Contigs/isotigs (*isotig_dataset*)	4845	18%	3903	11%	5144	17%
Singletons	22,807	82%	30,743	89%	24,645	83%
Total bases (Mbps):	15.8		20.1		17.1	

For all datasets, there was significant mitochondrial transcription (4.6–7.5%), as revealed by transcriptome mapping to the *T. dimidiata* mitochondrial genome [22]. The *T. dimidiata* coverage of the mitochondrial genome was near complete, whereas for *T. pallidipennis* and *T. infestans* 86% and 46% of the mitochondrial genome was covered, respectively (Table 2). Nevertheless, mitochondrial coverage in the three species was sufficient to allow the identification of all the mitochondria-encoded genes involved in oxidative phosphorylation. Only in *T. infestans*, there was significant transcription (13 K reads, 2.4%) that covered 100% of the reported *Triatoma* virus genome sequence (Table 2).

**Table 2:** Transcriptome mapping

	*T. dimidiata*		*T. infestans*		*T. pallidipennis*	
Match to *R. prolixus* genome (BLASTN)^b^		%		%		%
Non-redundant *Triatoma* transcripts matching *R. prolixus*	17,105	61.9%	18,074	52%	17,824	60%
Isotigs/contigs^a^	3,544	73.1%	2,459	63%	3,802	74%
Singletons	13,561	59.5%	15,615	51%	14,022	57%
Match to *R. prolixus* proteome (BLASTX)^c^						
*R. prolixus* proteins (best-hit)	7,865	52%	7,272	48%	8,002	53%
Pair wise identity		78%		79%		79%
*R. prolixus* proteins (e-value < 1.0E-05)	11,136	74%	10,674	71%	10,921	72%
*Triatoma* transcripts matching R. prolixus (e-value < 1.0E-05)	17,576	64%	18,836	54%	18,245	61%
BUSCO^d^	2,297	85.8%	2,201	82.2%	2,326	86.9%
CEG^e^	432	94.3%	415	90.6%	436	95.2%
*T. dimidiata* Mitochondrial genome						
Mapped reads	26,670	7.4%	25,714	4.6%	47,131	7.5%
Coverage (bp)	16,309	95.8%	7,937	46.6%	14,675	86.2%
Triatoma virus						
Mapped reads	31	0.01%	13,301	2.4%	8	0.0%
Coverage (bp)	3,234	35.8%	9,012	100.0%	1303	14.4%

^a^Contigs in non-redundant dataset

^b^Rhodnius-prolixus-CDC_SCAFFOLDS_RproC3.fa

^c^Rhodnius-prolixus-CDC_PEPTIDES_RproC3.1.fa . 15,078 proteins

^d^hmmsearch score ≥ 40; *n* = 2676 BUSCO’s. *R. prolixus* 2597/2676. 97.0%

^e^hmmsearch score ≥ 40; *n* = 458 CEG’s. *R. prolixus* 448/458. 97.8

### Comparison to other proteomes

The number of *Triatoma* transcripts that best-matched to the *R. prolixus* predicted peptide dataset were similar among the three species (~ 7272 to 8002), which corresponds to 48–53% of the *R. prolixus* predicted proteome (Table 2). Between 71 and 74% of the *R. prolixus* proteome had a match (e-value < 1.0E-05) in the *Triatoma* transcriptomes. Transcriptome completeness assessment by searching the Core Eukaryotic Genome Dataset (CEGMA) [23] and the Benchmarking Universal Single Copy Orthologs (BUSCO) [24] for the three - *Triatoma* and *R. prolixus* revealed very high coverage values. The BUSCO coverage was 82.2% in *T. infestans*, 85.8% in *T. dimidiata* and 86.9% in *T. pallidipennis,* while these coverage values were higher than 90% for the CEGMA in the three species (Table 2). These metrics indicate that although our datasets do not comprise all the gene content of each species, it is sufficient for a useful approximation for transcriptome to genome gene content comparison.

Each *Triatoma* transcriptome covers between 20 to 55% of the proteome of other insects (Table 3). Low proteome coverage was observed for *Drosophila melanogaster,* with a high-quality annotated proteome, *Ixodes scapularis,* and *Acyrtosiphon pisum.* BLAST best-hit protein identity was on average around 55% and correlated with the phylogenetic distance, such that average protein identity between the three - *Triatoma* and *R. prolixus* was ~ 78% (Table 2).

**Table 3:** BLASTP comparison of the three *Triatoma* translated transcriptomes with arthropod proteomes

	*T. infestans*				*T. pallidipenis*				*T. dimidiata*				
	*Triatoma* hits	Ref. hits	Ref. coverage (%)	Identity (%)	*Triatoma* hits	Ref. hits	Ref. coverage (%)	Identity (%)	*Triatoma* hits	Ref hits	Ref. coverage (%)	Identity (%)	Reference size (Pb)
*P. papatasi*	9851	4493	40	54	9966	4756	43	54	9602	4650	42	53	11,175
*P. humanus*	10,859	5100	47	57	12,066	5486	51	57	11,576	5384	50	56	10,788
*G. morsitans*	9975	4629	37	55	10,887	5000	40	54	10,461	4884	39	55	12,553
*An. gambiae*	10,357	5260	35	56	11,508	5647	38	55	11,074	5519	37	55	14,870
*C. quinquefasciatus*	10,588	5255	28	55	11,470	5666	30	55	10,955	5522	29	55	19,032
*Ae. aegypti*	10,398	5314	31	56	11,526	5732	33	55	11,099	5569	32	55	17,158
*Ac. pisum*	12,869	6222	17	54	12,416	6129	17	55	11,826	5958	16	55	36,195
*L. longipalpis*	10,109	4203	42	53	10,460	4493	44	53	9994	4413	44	53	10,110
*I. scapularis*	8883	4432	22	53	9537	4782	23	52	9147	4629	23	52	20,486
*D. melanogaster*	9966	5684	19	56	11,127	6093	20	55	10,718	5953	20	54	30,277

### Orthologous genes within *R. prolixus* and *Triatoma*

**Fig. 1:**
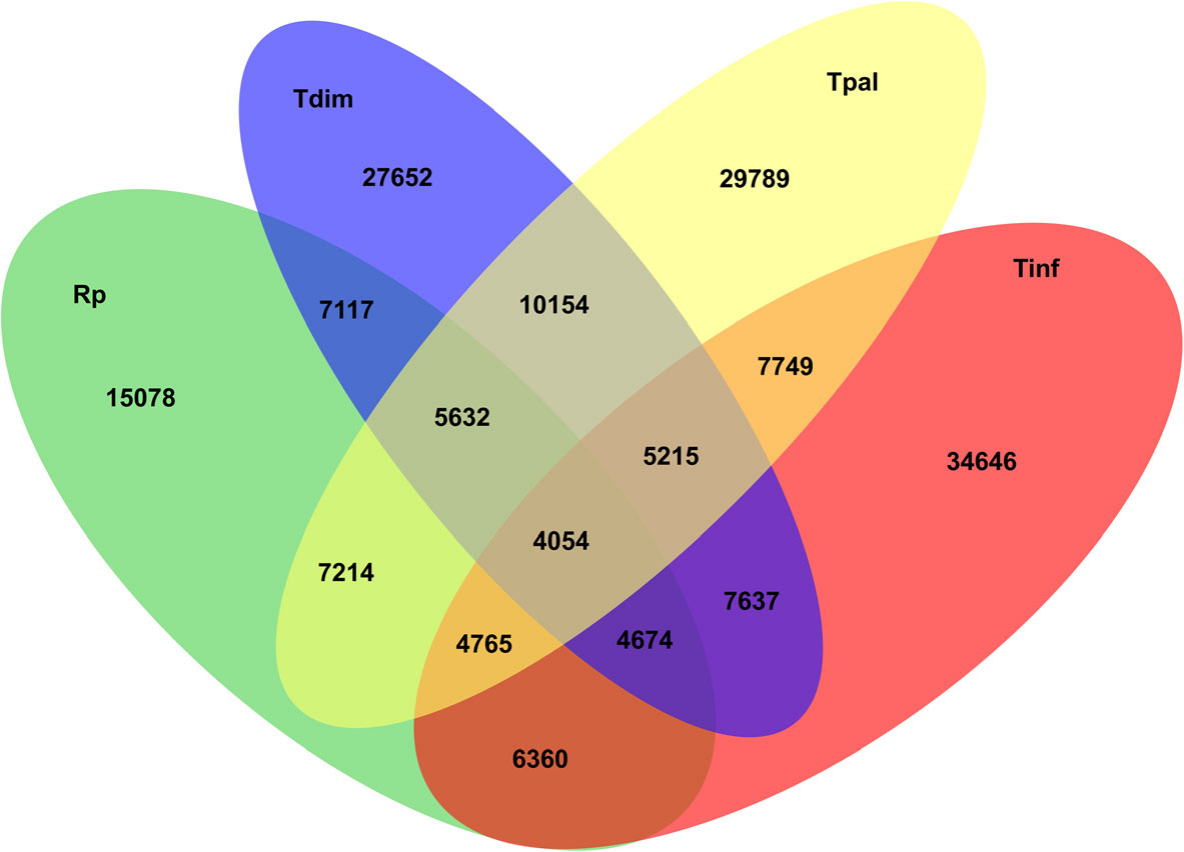
Putative orthology search within *Triatoma* and *R. prolixus*. Bidirectional BLASTX and TBLASTN searches were performed between all four datasets, using the BLAST best reciprocal hit strategy to define putative orthologs. The numbers in the non-overlapping areas correspond to the total number of transcripts in each species. We identified 4054 1:1 orthologs in the four species. Whereas *T. pallidipennis* and *T. dimidiata* shared more than 10 thousand 1:1 orthologs, they shared around 7600 with *T. infestans*, recapitulating species phylogeny

A total of 4054 orthologs were shared (BLAST best reciprocal hits) among the three - *Triatoma* and *R. prolixus*. A set of 5215 orthologs were present only in the *Triatoma* sp. studied here. In accordance to the phylogenetic distance, *T. dimidiata* and *T. pallidipennis* had 10,154 best BLAST reciprocal hits, while the best reciprocal hits among the other triatomines was lower (Fig. 1).

### Gene ontology (GO) annotation

The functional composition of each transcriptome can be influenced by biological variables (tissue type, ontogenic stage, gene content, genetic variation, transcription rate, among others), as well as for technical variables (tissue composition in the library, sequencing depth, normalization process, among others). To assess transcriptional representation bias, we tested Gene Ontology annotated transcriptomes for enrichment compared to the *R. prolixus* predicted proteome. Despite library normalization, the *Triatoma* transcriptomes were consistently enriched for transcripts involved in core cellular functions, such as protein translation, folding and sorting, generation of precursor metabolites and energy, mitochondrial and ribosomal organization and assembly (Table 4). In contrast, transcripts related to cell signaling, neurological processes, transcription and cell surface proteins were underrepresented in all *Triatoma* transcriptomes.

**Table 4:** GO enrichment in the 3 Triatoma transcriptomes in reference to *R. prolixus*. fisher exact test. FDR < 0.05

GO-ID	Term	Category	Over/Underepresented
GO:0005739	mitochondrion	CC	OVER
GO:0005783	endoplasmic reticulum	CC	OVER
GO:0005829	cytosol	CC	OVER
GO:0005840	ribosome	CC	OVER
GO:0043234	protein complex	CC	OVER
GO:0003729	mRNA binding	MF	OVER
GO:0003735	structural constituent of ribosome	MF	OVER
GO:0008135	translation factor activity, RNA binding	MF	OVER
GO:0019843	rRNA binding	MF	OVER
GO:0030234	enzyme regulator activity	MF	OVER
GO:0051082	unfolded protein binding	MF	OVER
GO:0006091	generation of precursor metabolites and energy	BP	OVER
GO:0006412	translation	BP	OVER
GO:0006457	protein folding	BP	OVER
GO:0006461	protein complex assembly	BP	OVER
GO:0006605	protein targeting	BP	OVER
GO:0007005	mitochondrion organization	BP	OVER
GO:0008219	cell death	BP	OVER
GO:0022618	ribonucleoprotein complex assembly	BP	OVER
GO:0042254	ribosome biogenesis	BP	OVER
GO:0044281	small molecule metabolic process	BP	OVER
GO:0044403	symbiosis, encompassing mutualism through parasitism	BP	OVER
GO:0051186	cofactor metabolic process	BP	OVER
GO:0005886	plasma membrane	CC	UNDER
GO:0001071	nucleic acid binding transcription factor activity	MF	UNDER
GO:0003677	DNA binding	MF	UNDER
GO:0004871	signal transducer activity	MF	UNDER
GO:0008168	methyltransferase activity	MF	UNDER
GO:0016301	kinase activity	MF	UNDER
GO:0043167	ion binding	MF	UNDER
GO:0007267	cell-cell signaling	BP	UNDER
GO:0050877	neurological system process	BP	UNDER

We next asked if there was biased transcriptional representation according to GO within the three - *Triatoma* transcriptomes. In the *T. infestans* transcriptome, we found overrepresentation of the GO term “symbiosis, encompassing mutualism through parasitism” (GO:0044403). No significant enrichment was found within the *T. dimidiata* and *T. pallidipennis* transcriptomes. The biased transcriptional representation found in the *Triatoma* datasets, using the *R. prolixus* as reference dataset, may result from transcriptional abundance of core cellular processes and the underrepresentation of certain organs/tissues, such as nervous system and sensory organs during library preparation, and may not reflect accurately gene content. Moreover, the lack of significant enrichment within *Triatoma* datasets indicates that the source of bias may be predominantly due to transcriptional abundance, and in a lesser extent to differences in gene content, with little participation of technical variation during library preparation.

### Protein family overrepresentation

To assess potential gene expansions, transcriptomes were annotated using InterProScan [25] through the BLAST2GO Pro interface [26]. 17.9, 24.2 and 22.8% of the respective *T. infestans*, *T. dimidiata*, *T. pallidipennis* non-redundant dataset, and 59.6% of the *R. prolixus* transcript dataset had at least one InterPro annotation. A Fisher’s exact test was performed to identify enrichment of InterPro entries in the test transcriptome *isotig* dataset (1 isotig per isogroup, excluding singletons) compared with the *R. prolixus* transcript dataset as reference. In some cases, significant enrichment (False Discovery Rate, FDR < 0.05) was not congruent with absolute gene number enrichment. To account for confounding effects due to transcriptome expression bias, emphasis was put on those InterPro entries in which absolute transcript counts were greater than in *R. prolixus* in at least one *Triatoma* species. Enrichment results are summarized in Table 5.

**Table 5:** Fisher’s Exact Test for InterPro domain enrichment

IPR id	Description	# Genes/transcripts^a^				Ref: *R. prolixus (FDR)*			Ref: *T. infestans*	
		RPRO	TINF	TDIM	TPAL	Ti-Rp	Td-Rp	Tp-Rp	Ti-Td	Ti-Tp
IPR005657	Triabin/Procalin	46	70	33	32	4E-32	4E-05	2E-04	2E-04	4E-06
IPR011038	Calycin-like	72	79	36	40	1E-30	2E-03	6E-04	2E-05	4E-06
IPR012674	Calycin	86	79	36	40	2E-27	2E-02	1E-02	2E-05	4E-06
IPR000618	Insect cuticle protein	84	35	27	17	2E-04	n.s.	n.s.	n.s.	4E-02
IPR003286	RNA-directed DNA polymerase	0	7	0	0	2E-04	n.s.	n.s.	n.s.	n.s.
IPR012336	Thioredoxin-like fold	79	30	38	34	4E-03	2E-03	n.s.	n.s.	n.s.
IPR006170	Pheromone/general odorant binding protein	29	17	13	19	4E-03	n.s.	5E-02	n.s.	n.s.
IPR002557	Chitin binding domain	46	21	10	12	7E-03	n.s.	n.s.	n.s.	n.s.
IPR008037	Pacifastin domain	9	9	12	17	3E-02	7E-03	6E-05	n.s.	n.s.
IPR000010	Cystatin domain	1	4	9	6	n.s	5E-04	5E-02	n.s.	n.s.
IPR020849	Small GTPase superfamily, Ras type	15	3	6	19	n.s	n.s.	2E-04	n.s.	n.s.
IPR000217	Tubulin	19	6	15	13	n.s	2.3E-02	n.s.	n.s.	n.s.
IPR001254	Serine proteases, trypsin domain	89	1	34	31	n.s	n.s	n.s	1E-04	2.0E-03

^a^Total number of InterPro annotated genes/transcripts: *T. dimidiata*: 1884; *T. infestans*: 1419; *T. pallidipennis*: 2110; *R. prolixus*: 10,407

#### Protease inhibitors and proteases

**Fig. 2:**
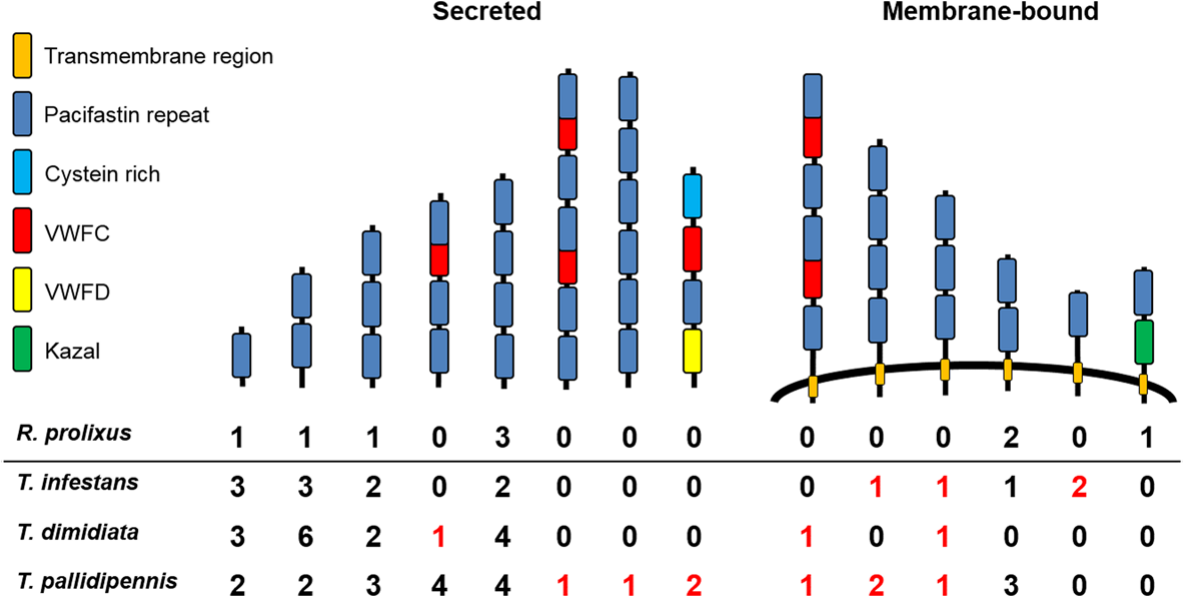
Structural characterization of the pacifastin family in the three - *Triatoma* species and *R. prolixus*. Based on InterPro annotations, transcripts of the *non-redundant* dataset (including singletons) were classified according to the presence of signal peptide, transmembrane regions and other InterPro domains. Absolute numbers for each type and species are shown in the matrix. Apart from the increase in gene number, particularly observed in *T. pallidipennis* and *T. dimidiata* (Table 4), we observed divergence and evolutionary innovation in secreted and membrane-bound pacifastins (red numbers). VWFC, von Willebrand Factor C domain; VWFD, von Willebrand Factor D domain. A detailed description of the pacifastin domain coding transcripts in Triatoma is found in Additional file 1

A numerical enrichment of transcripts coding for protease inhibitor domains of the pacifastin (*T. dimidiata* and *T. pallidipennis*) and cystatin (the three species) families, was identified (Table 5). *T. infestans* and *R. prolixus* had nine transcripts/genes encoding pacifastin domains (IPR008037), which corresponded to a statistical enrichment in *T. infestans* (FDR 3.0^− 2^) (Table 5), although the validation of a pacifastin expansion awaits the availability of *T. infestans* genome sequencing. Pacifastins are a family of protease inhibitors belonging to the MEROPS inhibitor family, involved in the regulation of different proteolytic cascades, including phenoloxidase-dependent melanization [27]. Pacifastin overrepresentation was more remarkable in *T. pallidipennis* with 17 unique transcripts compared with nine genes in the *R. prolixus* genome (FDR = 6.0^− 5^). Domain architecture analysis revealed novel domain configurations in *T. pallidipennis* absent in *R. prolixus* predicted proteome such as a pacifastin with von Willebrand factor, type C (IPR001007) and D domains (IPR001846), as well as a membrane-anchored two pacifastin-repeat coding transcript, and a six pacifastin domain transcript encoding a putative secreted product (Fig. 2). A membrane-anchored pacifastin containing a Kazal-type protease inhibitor domain (IPR002350) present in *R. prolixus* was not found in the three - *Triatoma* species (Fig. 2). A detailed characterization of pacifastin domain-encoding transcripts in the *non-redundant datasets* is in Additional file 1.

Transcripts encoding for InterPro domains belonging to protease inhibitors other than pacifastins were also more abundant. Cystatins are important regulators of cathepsin-mediated intracellular proteolysis [28, 29]. There is only one cystatin domain-type (IPR000010) protease inhibitor gene in *R. prolixus*, but cystatins were more abundant in *T. dimidiata* (*n* = 9), *T. pallidipennis* (*n* = 6), and *T. infestans* (*n* = 4).

Serine-type protease gene expansions are common in hematophagous arthropods [14, 30]. Enrichment of trypsin-domain serine proteases (IPR001254) was not found in any of the three Triatoma species. However, serine proteases were numerically and statistically depleted in *T. infestans*, regarding *T. dimidiata* (FDR = 1.0^− 4^) and *T. pallidipennis* (FDR = 2.0^− 3^).

#### Calycins

Calycins belong to a large family of extracellular proteins involved in a variety of functions, including binding of lipophilic compounds. A structurally related sub-family, the triabin/procalin family is an important component of hematophagous insects, including *Triatoma*, and function as anti-hemostatic factors [20, 31]. Transcripts containing the calicyn fold (IPR012674) were statistically enriched in the three - *Triatoma* species (Table 5). In particular, the calycin-like fold were enriched in *T. infestans* (*n* = 79), compared to *R. prolixus* (*n* = 72) (FDR = 1.0^− 30^). This calycin enrichment can be attributed to a significant triabin/procalin (IPR005657) expansion in the three species, but again higher in *T. infestans* (*n* = 70 versus 40 in *R. prolixus*. FDR = 4.0^− 32^). As expected, the calycin fold was also enriched in *T. infestans* over *T. pallidipennis* (*n* = 40, FDR = 4.0^− 6^) and *T. dimidiata* (*n* = 36, FDR = 2.0^− 5^). Calycins are classified according to the GO biological process “symbiosis, encompassing mutualism through parasitism” (GO:0044403), explaining the corresponding GO term enrichment in *Triatoma* sp., particularly in *T. infestans* (Table 5).

**Fig. 3:**
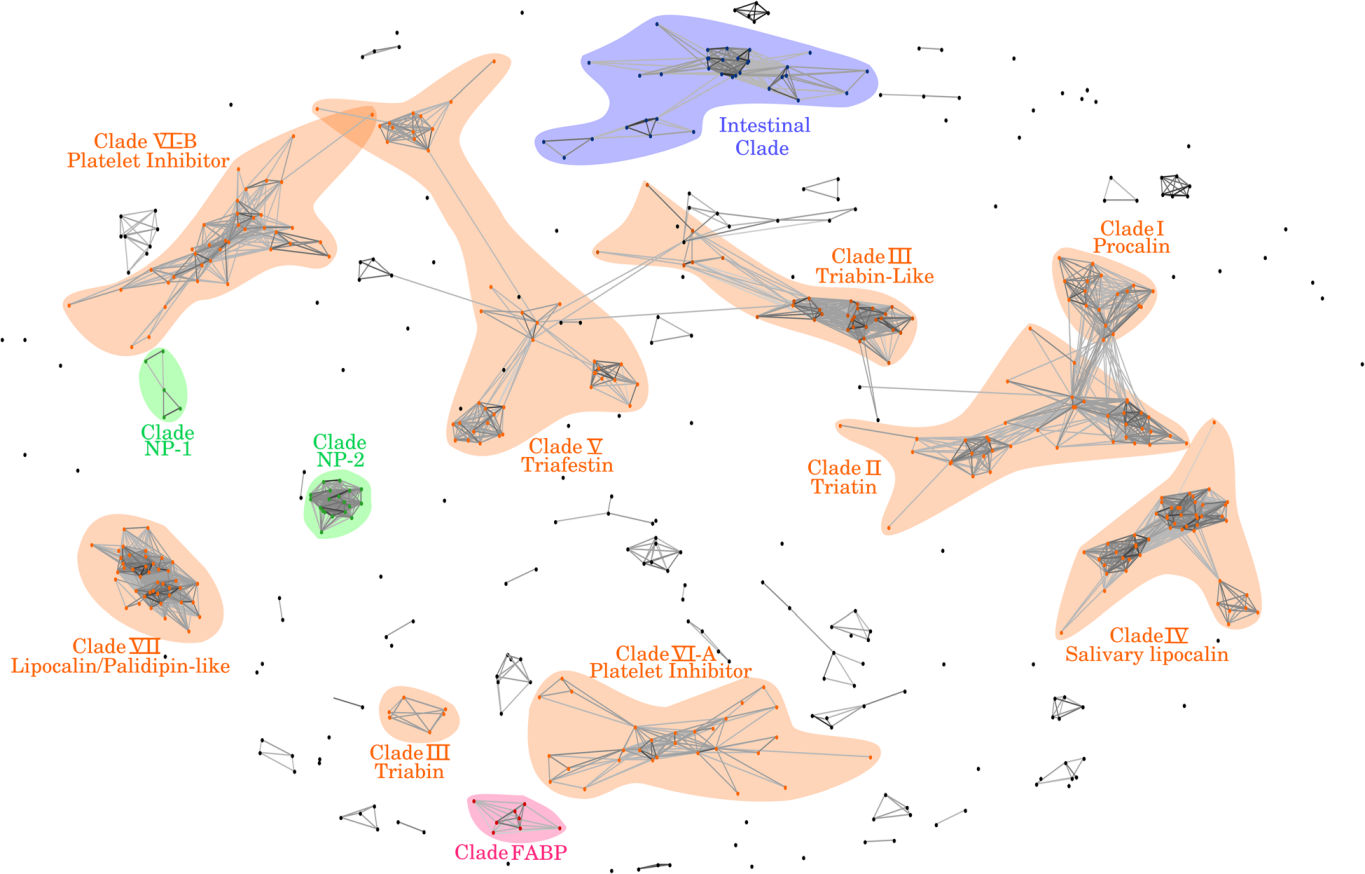
Clustering analysis of the Calycin/lipocalin gene family with CLANS in the three-*Triatoma* and *R. prolixus*. 2D representation of the clusters obtained through the CLANS analysis. A large calycin expansion was identified in the three-*Triatoma* species, but more remarkable in *T. infestans*. Although such expansion involves typical salivary lipocalins (clades I, II, III, IV, V, VI and VII, and nitrophorins) two novel non-salivary clade were identified: the Intestinal clade (purple) and the FABP (pink). The colored dots in the graph shown indicate sequences included in each calycin group, while black dots represent divergent sequences that do not cluster within any predefined clade. A detailed description of the calycin coding transcripts in *Triatoma* is found in Additional file 2

Our transcriptome datasets are consistent with the high structural complexity of the calycin family. To characterize the calycin types and their functions that were expanded in the three - Triatoma species, we followed a pair-wise comparison clustering approach, which included previously identified metazoan calycins along with those identified in our datasets. Through CLANS clustering analysis [32], we identified 11 major clusters in our *Triatoma* transcriptomes that corresponded to procalins (clade I), triatins (clade II), salivary lipocalins (clade IV), triafestins (clade V), collagen-induced platelet aggregation calycins (clade VI), and lipocalins/palidipin-like proteins (clade VII) [31]. However, we did not identify any procalin orthologs in *T. infestans,* and triabins - clade III calicyns in our *Triatoma* transcriptomes (Fig. 3).

In addition, we found several *T. infestans, T. dimidiata* and *T. pallidipennis* contigs sharing high sequence identity with nitrophorins, salivary heme-containing proteins, which participate in nitric oxide (NO) transport and storage [33]. These transcripts clustered with recently classified *R. prolixus* nitrophorins (RPRC000537 and RPRC000163) (green clusters, Fig. 3) [14], but not with the *R. prolixus* nitrophorins NP1-NP4.

One interesting finding from the clustering analysis was the identification of two clusters that lacked salivary calycins in the three –*Triatoma* species. In one of them, many sequences clustered with previously identified lipocalins in the intestinal transcriptome of *R. prolixus* [34] (purple clade, Fig. 3), referred here after as the intestinal clade. In the other group, sequences clustered with two *R. prolixus* sequences and fatty-acid binding proteins (FABP) highly conserved in insects and vertebrates (pink clade, Fig. 3). A detailed description of the three - *Triatoma* calicyn family clusters is provided in Additional file 2.

#### Transposable elements

**Fig. 4:**
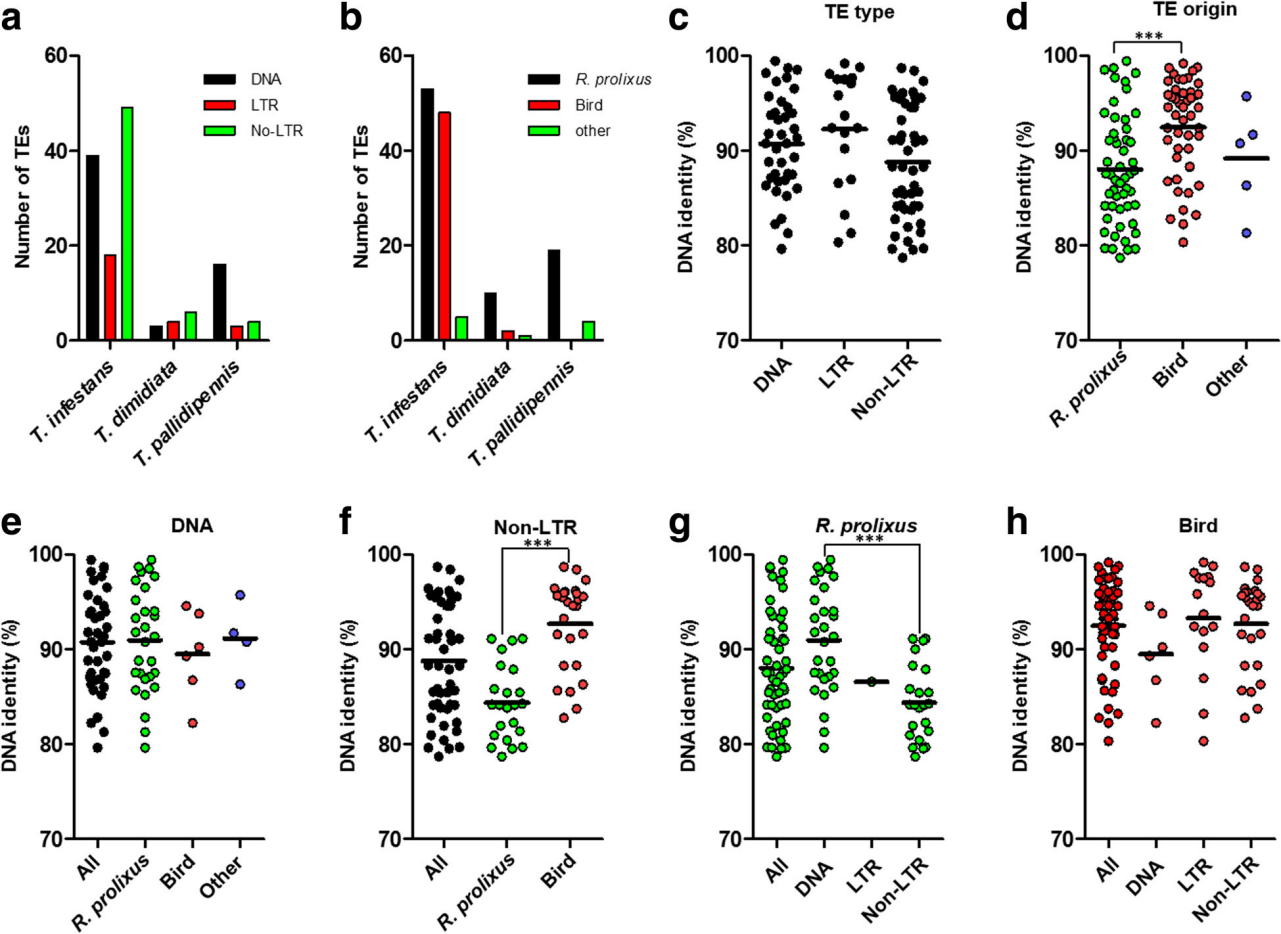
High transposable element (TE) transcriptional activity in *T. infestans****.*** **a** Absolute number of TEs classified according to type, and **b**) species of origin for the three - *Triatoma* species. For *T. infestans* only, **c**) DNA sequence identity (%) for each TE best-match according to TE type, and **d**) according to TE taxonomic origin. **e)** DNA sequence identity (%) in DNA TE best-match according to taxonomic origin. **f)** DNA sequence identity in LTRs best-match according to taxonomic origin. **g)** DNA sequence identity in *R. prolixus* best-match TEs according to TE type. **h)** DNA sequence identity in bird best-match TEs according to TE type. The increase in the absolute number TE in *T. infestans* is due to putative bird-derived non-LTR TEs. Higher sequence conservation suggests recent horizontal transfer; possibly form chicken, an important source of blood for *T. infestans*. A Kruskal-Wallis test with Dunn’s multiple comparison test was performed (*** *p* < 0.001). A full description of TEs analysis is in Additional file 3

Nine unique transcripts containing the RNA-directed DNA polymerase domain (IPR003286) were found in *T. infestans*, but none in *R. prolixus* and the other *Triatoma* datasets. A detailed mapping against the RepBase database [35] revealed a marked increase in the absolute numbers of transcriptionally active Long Terminal Repeat (LTR), non-LTR retrotransposons, and DNA transposable elements in *T. infestans* (*n* = 106), compared with *T. dimidiata* (*n* = 13) and *T. pallidipennis* (*n* = 23) (Fig. 4a, Additional file 3). The majority of *T. infestans* TEs were non-LTRs (46%), mainly CR1, *Jockey, Nimb* and *Loa*-type; followed by DNA TEs (36%), mainly *Mariner*-like. There was at least a five-fold increase in the number of LTRs in *T. infestans* (16% of all TEs), mainly ERV-type. In *T. pallidipennis,* the majority were DNA TEs (69%), being Mariner-like the most common (Additional file 3).

Although half of TEs identified in *T. infestans* best matched with *R. prolixus* TEs, a large proportion (45%) best matched to bird TEs (Fig. 4b and d). These sequences were mainly CR1-like non-LTRs and ERV-like LTRs (Additional file 3). Nucleotide sequence identity of *T. infestans* non-LTRs (CR1-like) with their corresponding bird best match was significantly higher than those best-matching *R. prolixus* non-LTRs (*Jockey, Nimb and Loa*) (Fig. 4d and f) and DNA TEs (Fig. 4g) (*p* < 0.001) (Additional file 3).

#### Olfaction gene families

A statistically significant enrichment of odorant binding proteins (OBPs) (IPR006170) was found in *T. pallidipennis* and *T. infestans* (Table 5). Because of their role in feeding preference and mating and other behavioral traits [36], we further searched for chemoreceptor-coding transcripts. We identified between 106 and 123 chemosensory genes distributed belonging to the most representative gene families among the three transcriptomes: odorant receptors (PF02949); ionotropic receptors (PF00060); gustatory receptors (PF08395); chemosensory proteins (PF03392); SNMP/CD36 (PF01130); mechanoreceptors (PF02949); and pickpocket receptors (PF00858) (Additional file 4). Taking into account putative orthologs (BRH between *Triatoma* and *R. prolixus*), OBPs and CSPs were the most abundant families. As for pacifastins in *T. infestans*, validation of the OBP family expansion awaits the availability of the full genome sequence of the corresponding triatomine.

#### Other protein families

An additional number of protein families/domains were enriched in the three -*Triatoma* species or in a particular species (Table 5). The number of Ras-type, small-GTPase superfamily (IPR020849) encoding transcripts was increased only in *T. pallidipennis* (*n* = 19 vs. 14 in R*. prolixus*; FDR = 2.0^− 4^). Other protein families were enriched only in statistical terms. The thioredoxin-like domain (IPR012336) was enriched in the three species; however, absolute numbers were lower than in *R. prolixus*. In *T. dimidiata*, there were 15 tubulin (IPR000217) transcripts compated to 19 in *R. prolixus* (FDR = 2.3^− 2^). Finally, statistical enrichment regarding *R. prolixus* of insect cuticle protein (IPR000618) (FDR = 2.0^− 4^) and chitin-binding domains (IPR002557) (FDR = 7.0^− 3^) was found *in T. infestans*, which might be implicated in cuticle formation, melanization and perimicrovilliar membrane formation [37–39]. As previously stated, validation of a gene expansion in these gene families awaits the availability of the full genome sequence of these triatomines.

### Protein divergence according to functional classes

**Fig. 5:**
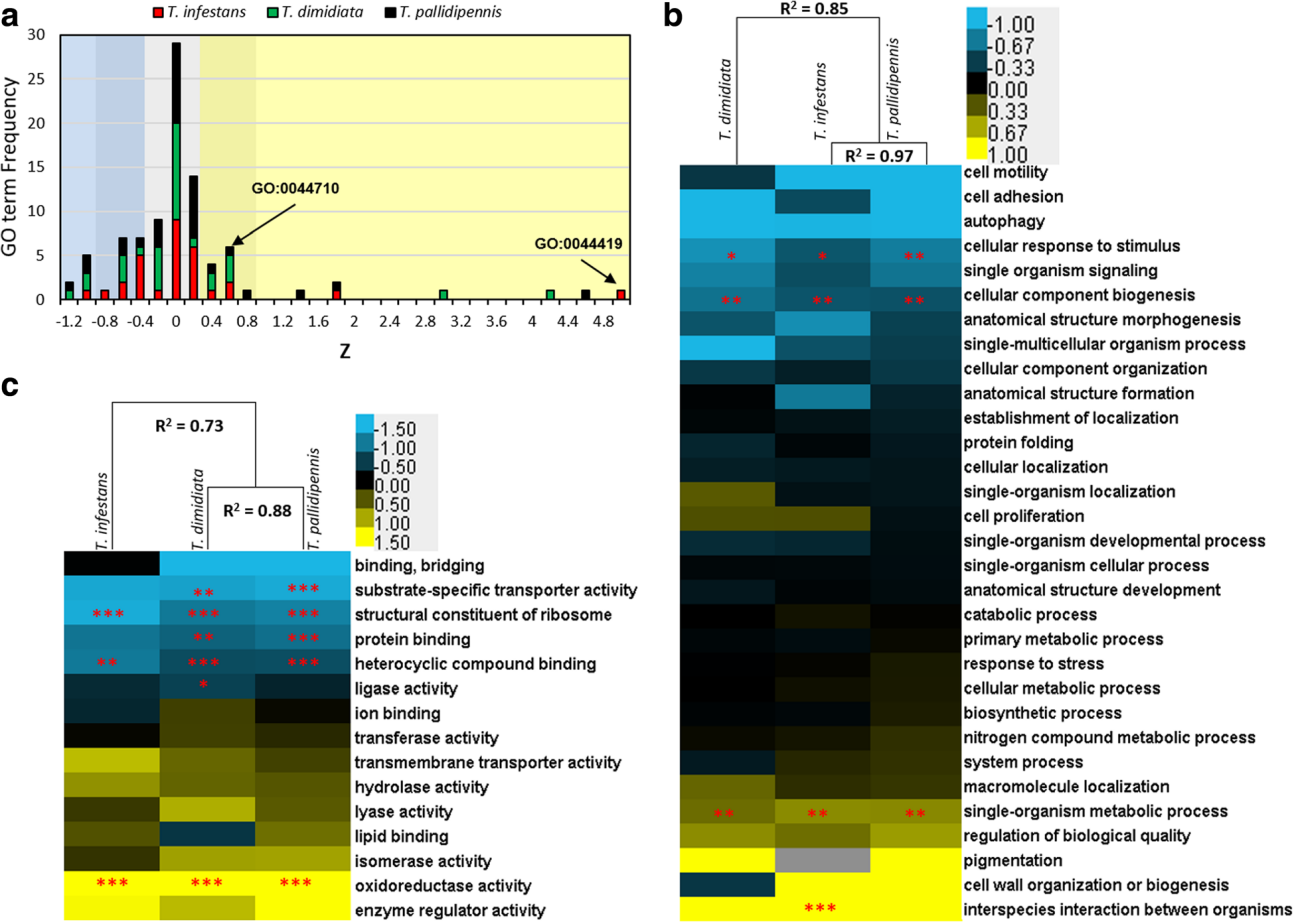
Structural divergence between the three - *Triatoma* 1:1 orthologs (BLAST best reciprocal hit to *R. prolixus*). **a** Distribution of GO (biological process) term frequency (*y* axis) according to Z value (the number of standard deviations from the median mean), as a measure of protein divergence (*x* axis). Negative Z values indicate higher conservation, whereas positive Z values indicate higher divergence. For instance, GO:0044710 (“single organism metabolic process”) has the same Z value in the three species, but is less divergent than GO:0044419 (“interspecies interaction between organisms”), with different although high Z values in the three species. **b** Biological process Z values for level 3 Gene ontology terms GO’s (*rows*). The Z value was numerically ordered according to *T. pallidipennis* and hierarchically clustered (Pearson’s correlation) according to species (*columns*). Blue tones reflect higher protein sequence % identity (i.e. more conserved), whereas yellow tones reflect lower % identity (more diverged). Significant higher protein sequence divergence were found in “single organism metabolic processes”, “interspecies interaction between organisms”, and oxidoreductase activity. **c** Molecular function Z values. Gene ontology terms level 3 GO’s. Kruskal-Wallis one Way ANOVA. Dunn’s correction for multiple testing. *** *p* < 0.001; ** *p* < 0.01. Non statistical significance in some conserved and divergent GO classes was probably because the lack of power due to low numbers of genes in those classes. Data corresponding to this figure, including % identity mean for each GO term and cellular compartment terms is in Additional file 5

To gain insight into protein family evolution in triatomines, we assessed protein sequence divergence according to GO class as a relative measure of evolutionary rate among each *Triatoma* and *R. prolixus* 1:1 orthologs. To compare protein sequence conservation within *Triatoma* species, Z values (number of standard deviations from the mean % identity) were used. The majority of GO classes were distributed around Z = 0 (i.e. average divergence), but few classes had negative Z values (more conserved than the average), while fewer classes had positive Z values (more divergent than the average) (Fig. 5a).

Z values according to GO biological process and molecular function were clustered, based on their Pearson’s correlation by species and represented in a pseudocolor heatmap (Fig. 5b-c. Additional file 5). Putative ortholog pairs belonging to core biological process such as “cell signaling”, “cellular component biogenesis and organization”, “autophagy”, “morphogenesis” and “cellular responses to stimulus” were more conserved among species (Fig. 5b, negative Z scores in blue), although only “cellular response to stimulus” (GO:0051716) and “cellular component biogenesis” (GO:0044085) were statistically significant (Kruskal-Wallis one Way ANOVA. Dunn’s correction for multiple testing, *p* < 0.05). A high proportion of orthologs belonging to this GO term participate in protein synthesis and different components of the translation machinery and cell signaling. In contrast, orthologs annotated as “single-organism metabolic processes” (GO:0044710) were significantly less conserved in the three - *Triatoma* species (Kruskal-Wallis one Way ANOVA. Dunn’s correction for multiple testing, *p* < 0.05). Only in *T. infestans,* orthologs annotated as “interspecies interaction between organisms” (GO:0044419) were significantly less conserved (Fig. 5b. Positive Z scores in yellow. Additional file 5). Calycins belong to this term, indicating that in addition their numerical expansion; they are evolving at a fast rate in *T. infestans*.

Consistently, the same analysis based on “molecular function” and “cellular component” GO’s revealed high protein coding sequence conservation in the three - *Triatoma* species for orthologs involved in protein synthesis and nucleic acid or nucleoside binding. In particular, orthologs belonging to “structural constituent of ribosome” (GO:0003735), “heterocyclic compound binding” (GO:1901363), and “localization in the ribonucleoprotein complex” (GO:1990904) were significantly conserved at the protein sequence level. In contrast, significant higher protein sequence divergence was observed in “oxidoreductases” (GO:0016491) in the three species (Fig. 5c, Additional file 5). Many of these oxidoreductases are involved in respiration and other metabolic processes, which is in agreement with the observed divergence in “single-organism metabolic process” (GO:0044710) (Fig. 5a-b). In addition, “enzyme regulator activity” (GO:0030234) was the most divergent molecular function term, although this finding was not statistically significant, probably due to small sample size. Pacifastins and other protease inhibitors belong to this category; suggesting that proteolysis regulation trait in *Triatoma* may be evolving at a high rate (Additional file 5).

Hierarchical clustering of protein sequence conservation (Z scores) according to biological process (Fig. 5b) and cellular component (Additional file 5) did not correlate with the corresponding *Triatoma* phylogenetic relationships, in which *T. pallidipennis* and *T. dimidiata* are more closely related than with *T. infestans.* However, clustering according to molecular function did recapitulate the established phylogeny. (Fig. 5c).

To identify genes/functions that show higher protein sequence divergence, we asked if the most divergent ortholog pairs were enriched in more specific GO terms. One-to-one ortholog pairs were ranked according to protein pairwise identity (% identity with BLASTx) and the whole ranked dataset was subdivided into equal terciles or groups (Additional file 6). Using the most conserved group as reference (Additional file 6, blue), GO enrichment analysis (Fisher’s Exact test) confirmed that orthologs involved in cellular metabolism, particularly in carbohydrate metabolism and oxidative phosphorylation were enriched in the most divergent group (Additional files 6 and 7. yellow). This included many oxidoreductases participating in the respiratory chain complexes (Additional file 7). It also confirmed high protein divergence in the calycin family in *T. infestans* (“evasion or tolerance of host defense response”, GO:0030682), and “peptidase inhibitor activity”, GO:0030414) in *T. pallidipennis*. Importantly, biological processes such as carbohydrate, ketone and lipid metabolism were enriched in the most divergent group, suggesting that the whole energy generation and storage pathway may be evolving at higher rates. Additionally, “serine-type endopeptidase activity” (GO:0004252) was enriched in the most divergent group only in *T. dimidiata* and *T. pallidipennis*, indicating that*,* in addition to a numerical expansion (Table 5), serine proteases may be evolving at a high rate in these species regarding *T. infestans*.

### Reconstruction of the carbohydrate metabolic pathways

**Fig. 6:**
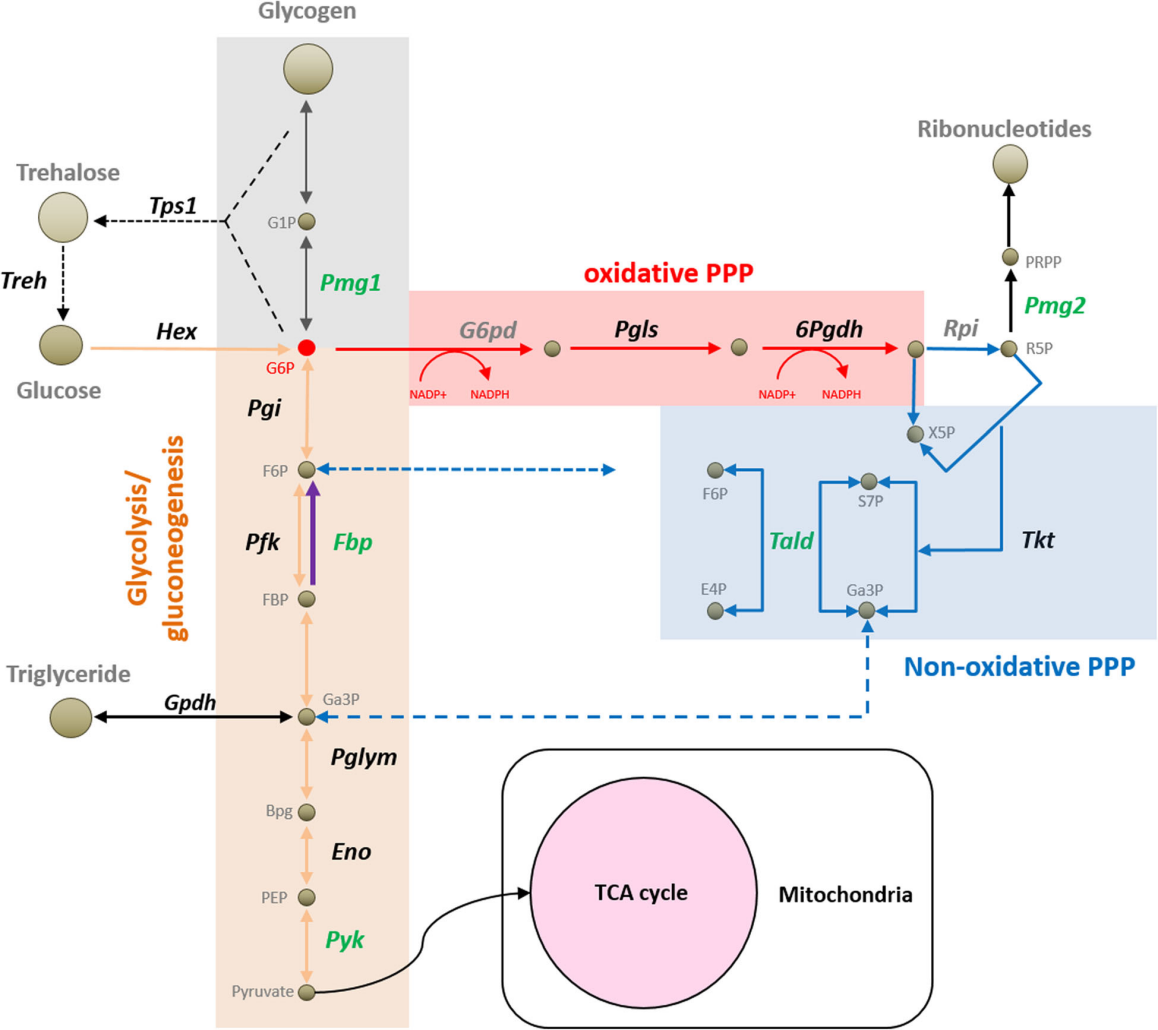
Schematic representation of metabolic enzymes belonging to the most divergent group (protein sequence identity) in the context of carbohydrate metabolism. Enzymatic mediators (***italics***) that were found in the divergent group in at least one *Triatoma* species (< 86% protein identity with putative *R. prolixus* orthologs, *Rprc3.2_mapped* dataset) are shown in *green*. Mediators that were not in the divergent group are in *black*, whereas those not found are labeled in *grey*. The glycolytic pathway is shown in *orange*. Gluconeogenesis is shown in *purple*, the oxidative phase of the pentose phosphate pathway is shown in *red.* The non-oxidative phase of PPP is shown in *blue*. Metabolites are shown in non italics *gray*. A full description of metabolic enzyme coding genes is described in Additional file 8

The 1:1 *Triatoma-R. prolixus* orthologs identified in the *RproC3.2_mapped* dataset involved in glycolysis/gluconeogenesis and pentose phosphate pathway (PPP) [14], as well as in threalose, glycogen and triglyceride metabolism were identified and labeled according to their protein divergence groups (Fig. 6. Additional file 8). Among 43 *R. prolixus* genes, orthologs for 36 were found in the three - *Triatoma* species, and the majority of these belonged to the intermediate or most conserved group. Only five orthologs involved in carbohydrate metabolism with coverage higher than 40% belonged to the most divergent group. A putative phosphoglucomutase 1 (*Pgm1*) ortholog, involved in glycolysis and glycogen synthesis was found in *T. pallidipennis,* but absent in *T. dimidiata* and *T. infestans* (Fig. 6). The *T. pallidipennis* ortholog for pyruvate kinase (*Pyk*) of the glycolytic pathway, fructose-1,6-bisphosphatase (*Fbp*) involved in gluconeogenesis was identified in the three – *Triatoma* species. Interestingly, only *Fbp* in *T. dimidiata* and *T. pallidipennis* belonged to the most divergent group, whereas the corresponding *T. infestans* ortholog belonged to the most conserved group (Fig. 6).

Among six *R. prolixus* genes encoding for PPP enzymes, only glucose-6-phosphate dehydrogenase (*G6pd*) was not found in the three - *Triatoma* species. A putative phosphoglucomutase/phosphopentomutase 2 (*Pgm2),* involved in purine and pyrimidine biosynthesis was found in *T. pallidipennis* and *T. infestans*, but only in the later belonged to the most divergent group (Fig. 6). The *T. pallidipennis* transaldolase (*Tald*) orthologs, linking the PPP to glycolysis, also belonged to the most diverse group (Fig. 6. Additional file 8).

### Divergence in oxidative phosphorylation

ATP production by oxidative phosphorylation is mediated by at least five mitochondrial multi-subunit complexes involved in electron transport, generation of a H+ gradient across the mitochondrial membrane and coupling such gradient to ATP synthesis. As for metabolic pathways, most of nuclear and all mitochondrial gene orthologs coding for the NADH dehydrogenase complex (I), succinate dehydrogenase complex (II), cytochrome B complex (III), cytochrome c oxidase complex (IV) and ATP synthase complex (V) were identified in *R. prolixus* and the three - *Triatoma* species. A significant proportion of subunits of complex I, IV and V belonged to the most divergent group (Additional file 9).

#### NADH dehydrogenase complex (I)

Complex I catalyzes the electron transfer from NADH to ubiquinone, as well as proton translocation into the inter-membrane space. The eukaryotic complex I core is composed by 14 subunits (seven encoded in the mitochondrial genome), an α subcomplex composed by 13 nuclear-encoded subunits, and a β subcomplex composed by 11 nuclear-encoded subunits. We identified 11 core subunits, 10 α subcomplex subunits, and all β subcomplex subunits in the three - *Triatoma* species and *R. prolixus*. All the mitochondria-encoded subunits, two and seven subunits of the α and β subcomplex, respectively belonged to the most divergent group (Additional file 9).

#### Succinate dehydrogenase complex (II)

Complex II participates in the TCA cycle and contributes as an electron donor to the ubiquinone pool. We identified all four subunits of complex II in the three *Triatoma* and *R. prolixus*. Only SdhD were in the most divergent group (Additional file 9).

#### Cytochrome B complex (III)

Complex III transfers electrons to cytochrome c from the ubiquinone pool and contributes to the proton gradient by translocating four H+. Nine of the 11 subunits of complex III were identified in the four species. Only two, QCR2 and QCR7 were in the most divergent group (Additional file 9).

#### Cytochrome oxidase C complex (IV)

In Complex IV, electrons in cytochrome c (complex III) are transferred to oxygen to form water, linked to the translocation of additional protons. Complex IV is composed by 20 subunits. Cox1, Cox2 and Cox3 are mitochondria-encoded. We identified 12 subunit orthologs, five of which were in the most divergent group in the three - *Triatoma* species (Additional file 9).

#### ATP synthase complex (V)

The ATP synthase couples the electrochemical proton gradient with the generation of ATP. It is composed of a F_O_ subcomplex, which constitutes the proton pore embedded in the inner mitochondrial membrane, and a F_1_ subcomplex, which mediates ATP synthesis. The F_O_ subcomplex is composed of at least eight subunits, two of which are mitochondria-encoded (ATP6 and ATP8). The F_1_ subcomplex is composed of five nuclear-encoded subunits (α, β, γ, δ and ε). Orthologs for the eight F_O_ subunits and all F_1_ were identified in the three - Triatoma and *R. prolixus*. All the components of the F_O_ subunit, but subunit C (pore-forming subunit), belonged to the most divergent group, while all F_1_ subunits were in the most or intermediately conserved groups (Additional file 9).

Other enzymes beyond complexes I-V participate in energy/redox metabolism, such as mitochondrial Glycerol 3-phosphate dehydrogenase (mG3P) and Proline dehydrogenase (ProDH), which may play an important role in insect vectors physiology [40, 41]. The corresponding mG3P orhtolog was found in *R. prolixus* and the three triatomas, although only in *T. dimidiata* it belonged to the most divergent group. In the case of ProDH, no orthologs were found in *T. dimidiata*, but singleton BRH’s were found in *T. infestans* in most divergent group, and in *T. pallidipennis* in the average conserved group (Additional file 9).

**Fig. 7:**
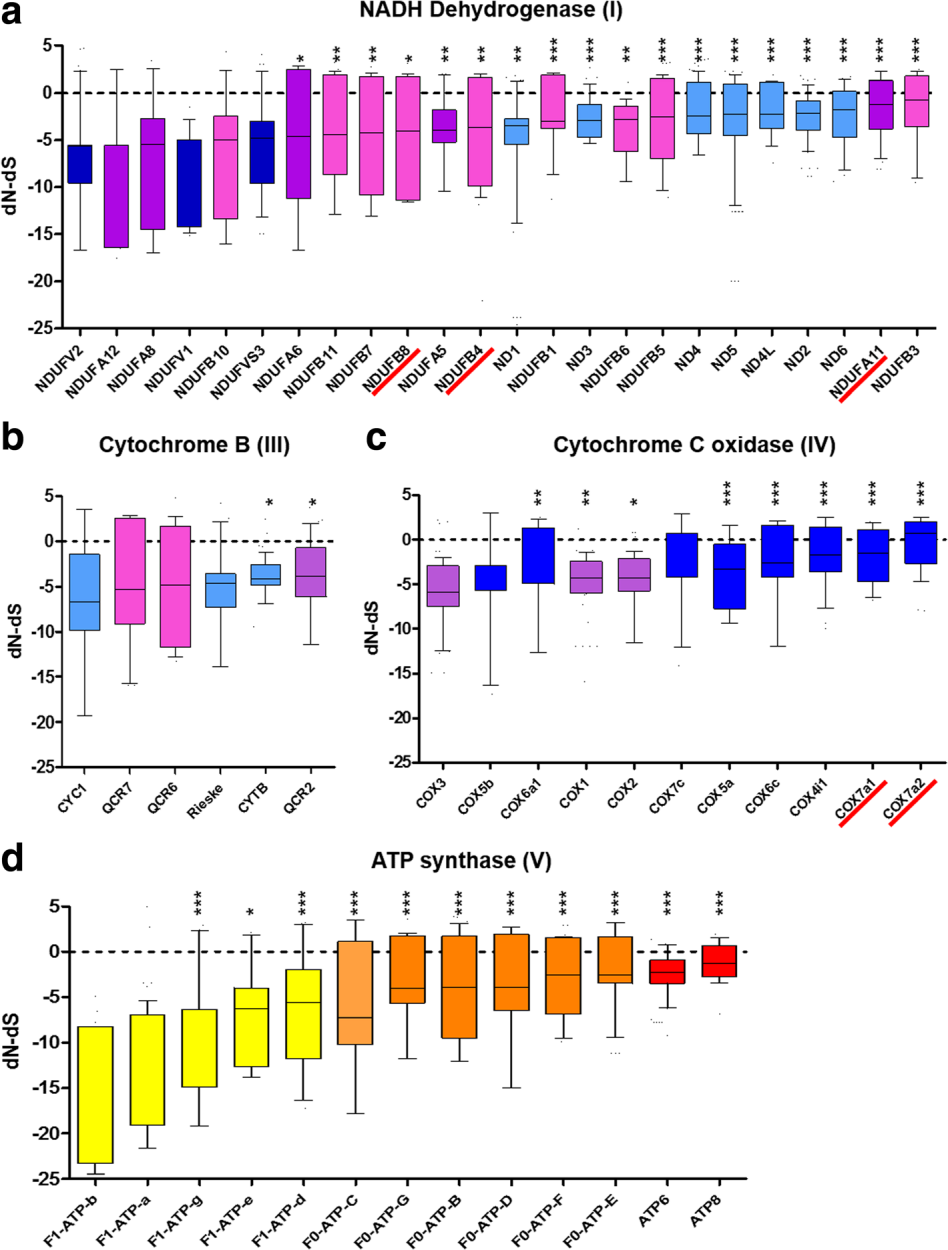
Distribution of normalized dN-dS in reduviid oxidative phosphorylation complex subunits. Codon-by-codon Maximum Likelihood analysis was calculated for each ortholog using the HyPhy package [42]. The normalized dN-dS for each variant position was plotted according to increasing median value from left to right. Positive values indicate likelihood of selection. **a** Complex I. Core mitochondrial subunits (*light blue*), core nuclear-encoded subunits (*dark blue*), accessory A subunits (*purple*), accessory B subunits (*pink*); **b** Complex III. Respiratory subunits (*light blue*), core subunit (*purple*), low molecular weight subunits (*pink*); **c** Complex IV. Mitochondria-encoded subunits (*purple*); and **d**) ATPase complex. F1 ATPase subunits (*yellow*), Nuclear-encoded F0-ATPase subunits (*orange*) and mitochondria-encoded F0 subunits (*red*). Significant differences (one-way ANOVA test corrected for multiple comparison) in dN-dS distribution are shown under black bar. Red arrows indicate subunits participate in the oligomerization of respiratory supercomplexes in mammals [77]. A full description of coding genes involved in oxidative phosphorylation is described in Additional file 9

We further tested if the genes coding for the oxidative phosphorylation complexes subunit orthologs were under positive (adaptive) selection in 23 orthologs of the NADH dehydrogenase complex (I), six of the cytochrome B complex (III), 12 of the cytochrome C complex (IV) and 13 of the ATP synthase complex (V) using MEGA7 (Kumar, et al. 2016). No evidence of positive selection (dN > dS) was found using a codon-based Z-test of positive selection (*p* > 0.05). Furthermore, we used the HyPhy package to estimate selection per codon, based on the difference of non-synonymous (dN) and synonymous (dS) substitutions maximum likelihood [42]. Positive normalized dN-dS values indicate higher likelihood that the codon is under positive selection (Fig. 7). Most codons presented negative normalized dN-dS values, which is consistent with negative selection. We detected positive normalized dN-dS values in many codons for several orthologs, suggesting positive selection, although these were not statistically significant. However, comparison of normalized dN-dS distribution of each ortholog revealed a significant bias towards positive normalized dN-dS values in six NADH dehydrogenase β subcomplex subunits, two α subcomplex subunits and all mitochondrial genome-encoded complex I core subunits (Fig. 7a). For complex III, significant differences in Cyt B and QCR2 dN-dS distribution was found (Fig. 7b). In contrast to mitochondrial-encoded subunits, several nuclear encoded subunits of cytochrome c oxidase complex, particularly COX7_A_, showed biased dN-dS distribution towards positive values (Fig. 7c).

In the case of the ATP synthase complex, consistent with the segregation of protein sequence divergence between the F_1_ and F_O_ subunits (Additional file 9), a remarkable segregation of dN-dS distribution was also detected. All components of the catalytic F_1_ subcomplex showed strong bias towards negative dN-dS values, whereas all F_O_ subcomplex subunits were significantly biased toward positive values (Fig. 7d).

## Discussion

We conducted a transcriptomic analysis of normalized cDNA libraries that cover the entire life cycle of three triatomine species relevant for Chagas disease transmission from Mexico to Argentina, *T. pallidipennis*, *T dimidiata* and *T. infestans*. To get insights into the triatomine biology, we followed a “data driven” research approach (gene content, protein family architecture, protein sequence divergence and positive selection inference) towards the identification of genetic signatures that could suggest adaptations to their particular and diverse life styles. From this approach, we expected to generate novel hypothesis regarding triatomine biology, rather than providing exhaustive characterization and hypothesis testing of genes involved in different biological processes.

Almost two-thirds of our de novo assembled datasets map to the *R. prolixus* genome and proteome, and three quarters of the *R. prolixus* proteome had a homologous match in the three - *Triatoma* datasets. Despite the relatively low throughput of the 454 sequencing technology compared with other sequencing technologies, library normalization contributed to the high coverage of the core eukaryotic genome (CEG) [23] and the core arthropod single-copy ortholog dataset (BUSCO) [24] (Table 2). Thus, the coverage estimation in our datasets sufficed for a meaningful analysis of the transcriptome and genome gene content comparisons. Despite normalization, genes expressed at very low levels or restricted to few cells could contribute to the incompleteness of the datasets. Gene family contraction and expansions are common during speciation and adaptation to ecological niches [43]; however we are aware that not all gene family expansions are adaptive [44]. Therefore, we focused on the analysis of gene family enrichment in absolute terms, which accurately reflect true gene expansions, and we suggest caution in the interpretation of those cases with statistical evidence, but no absolute numeric gene increase data. Deep transcriptome sequencing analysis of less abundant cell types, as well as full genome sequencing of the corresponding *Triatoma* species will be required for definitive genome content comparisons among reduviids.

InterPro annotation enrichment in the triatomines was assessed using *R. prolixus* as reference (Table 5). Remarkably, two types of protease inhibitors (PI) were numerically enriched in the triatomines: pacifastins and cystatins. Pacifastins were expanded at least in *T. dimidiata* and *T. pallidipennis*. Pacifastins were described in crustaceans as serine protease inhibitors that regulate the phenoloxidase cascade [45]. They are widely distributed in arthropods and almost all insect orders [27, 46]. Pacifastins were described in *T. infestans* sialotranscriptome [47], and an up-regulated pacifastin upon blood meal and bacterial challenge, was described in *T. infestans* fat body [48], suggesting that pacifastins may be important regulators of hematophagy and immune response in triatomines.

In the three – *Triatoma* species, we found no evidence of the Kazal domain containing-pacifastin present in *R. prolixus*, but its absence in *Triatoma* sp. cannot be ruled out. Although, our triatomine pacifastin architecture reconstruction may be incomplete due to the possibility of non-full length transcripts, the identification of novel domain configurations (Cystein-rich, VWFC and VWFD domains), particularly in membrane bound pacifastins (Fig. 2, Additional file 1), indicates evolutionary innovation in this protein family and supports the current view of adaptive diversification in the pacifastin family [46].

Cystatins belong to a large and widespread family of cysteine proteases (i.e. papain, cathepsins) inhibitors in plants, prokaryotes and animals [29]. Cystatins also play an anti-hemostatic role during blood meal in ticks [49, 50]; but they appear to be absent in the *Triatoma* sialotranscriptomes described so far [15–17, 31, 47, 51]. Tigutcystatin, a secreted midgut cystatin, may be involved in blood digestion and host - *T. cruzi* interaction in *T. infestans* [52], thus the cystatin expansion we documented in *T. pallidipennis* and *T. dimidiata* deserves further study to elucidate its biological significance. Taken together, gene expansions in two protein-inhibitor unrelated families (Table 5), with different cellular localization, substrate specificities and higher evolutionary rates (Fig. 5, Additional file 5), suggest high evolutionary dynamics in proteolysis regulation in triatomines. This includes, but is not limited to, adaptations in hematophagy, immune response and parasite-vector interactions.

A calycin/lipocalin lineage specific expansion was described in *R. prolixus* [14]. We found a significant enrichment in the three – *Triatoma* species, suggesting a gene family expansion that occurred in Triatomini after divergence from Rhodniini. Calycins form a large protein superfamily divided into lipocalins, fatty acid binding proteins, triabin, and thrombin inhibitors [53]. Of these, the lipocalin family (IPR002345) is the largest and functionally the most diverse. These are extracellular proteins sharing several recognition properties, such as ligand binding, receptor binding and the formation of complexes with other macromolecules, namely lipophilic compounds. Calycins constitute a preponderant component of several reduviid sialotranscriptomes, and are implicated in vasodilatation, anti-hemostasis and immunomodulation [15–18, 20, 31, 47, 51].

Lipocalin expansion was remarkable in *T. infestans*, where salivary lipocalins (clade IV), triafestin-like (clade V) and lipocalin/palidipin-like (clade VII) calycins groups doubled the numbers in *T. pallidipennis* and *T. dimidiata* (Additional file 2). Interestingly, we identified novel calycin groups, namely the intestinal and FABP clades (Fig. 3). Presumably, these novel calycins may be expressed in other organs or tissues than the salivary gland. A single FABP lipocalin, found in each analyzed *Triatoma*, is conserved in almost all metazoans and has been implicated in the day/night cycle modulation in *D. melanogaster* [54]. This opens the path for further structural, phylogenetic and functional characterization of the calycin/lipocalin family. The rapid expansion of calycins in *Triatoma,* as well as their higher evolutionary rates (Fig. 5), is consistent with an adaptive role of this protein family in blood feeding and possibly other relevant biological processes beyond those in salivary gland.

An important difference among the three - *Triatoma* species was the higher number (5 to 8-fold increase) of transcriptionally active transposable elements in *T. infestans*, particularly non-LTR retrotransposons (8 and 12-fold increase) (Fig. 4a)*.* This may be partially related to *T. infestans* genome size, which is one of the largest among all analyzed reduviids [55]; and genome size is associated with increased transposable and other repeated elements activity and content [56]. According to their putative taxonomic origin in RepBase database [35] and based on sequence identity, the majority of the non-LTR retrotransposons in *T. infestans* best-matched with bird CR1 non-LTR and showed higher sequence conservation than non-LTRs best-matching *R. prolixus* (*Jockey*, *Nimb* and *Loa*- type) (Fig. 4f). Horizontal transfer (HT) of DNA transposons from the blood meal source was recently proposed in *R. prolixus* [57]. Non-LTR HT is considered rare because it depends on unstable RNA intermediates. Nevertheless, evidence for non-LTR HT, including CR1 elements was documented in arthropods [58–60]. Also, endosymbiont to host genome gene transfer was demonstrated in the pea aphid *A. pisum* [61] and *R. prolixus* genomes [14]. Taken together, our results are consistent with recent HT events in *T. infestans* from birds (its major source of blood)*.* Although we cannot rule out that non-LTR transcripts could derive from chicken blood used for feeding, these were not found in *T. dimidiata* and *T. pallidipennis* transcriptomes, which were fed from the same blood source under the same conditions. Further research and the availability of the *T. infestans* genome will contribute to clarify this issue.

Aiming at identifying signs of molecular adaptation, we first used protein sequence identity in *Triatoma*-*R. prolixus* 1:1 orthologs as an indicator of differences in evolutionary rates. Clustering of divergent proteins in a biological pathway, a function or a biological process is suggestive of high evolutionary rates of that particular process or function, and could guide the search for molecular adaptation. To document adaptation, a directed test for selection is required. In our work, an important limitation of using these approaches was that in some cases, the full ORF for each pair was unavailable. Nevertheless, the analysis of protein sequence conservation based on Z score comparisons and enrichment in the most divergent group suggest high evolutionary rates in oxidoreductase-mediated central metabolism, protease inhibitors, serine proteases in the three Triatomini, and in the calycin family at least in *T. infestans*, (Fig. 5, Additional file 7). In the case of serine proteases, this is a common finding in hematophagous insects [14, 30, 62], however high sequence divergence in immune response genes was also expected, but not detected. Lower evolutionary rates in core cellular processes such as protein biosynthesis, transport, signaling are consistent with other comparative genomics studies in insects in general and hematophagous insects in particular [30]. The finding that the protein conservation pattern in biological processes and cellular components do not correlate to phylogeny (Fig. 5b-c) may reflect that molecular function has a stronger correlation with structural ancestry (Fig. 5c), whereas biological process and cellular component depend on the convergence of multiple genes with different structural ancestry.

In *D. melanogaster* and other insects, gene adaptations in critical enzymes determining carbohydrate metabolic flux depend on their life history, latitudinal clines and flight capabilities [63, 64]. Although most of the glycolytic pathway enzymes were highly conserved in the three - *Triatoma* species, we identified sequence divergence in enzymes involved in anabolism, such as gluconeogenesis (*Fbp*), glycogen synthesis (*Pgm1*) and the PPP (*Pgm2, Tald*); and one enzyme that participates in glycolysis (*Pyk*) (Fig. 6, Additional file 8). *Fbp* generates fructose 6-phosphate a crucial regulator of metabolic flux towards the PPP. The opposite reaction is mediated by the 6-phosphofructokinase, which is critical for glycolysis required by flight muscles in insects. Additionally, the simultaneous activity of both enzymes is implicated in a “futile cycle”, which generates heat from ATP hydrolysis, allowing the insect adaptation to temperate climates [65, 66]. Molecular adaptation in *Fbp* and its relation to flight loss in *Triatoma* is a hypothesis worth of further investigation, especially in *T. infestans*, which is subjected to extreme day/night temperature variations.

*Pgm1* is a critical enzyme catalyzing the interconversion of glucose 1-phosphate to glucose 6-phosphate, a critical step in the initiation of glycolysis and glycogen synthesis. Glycogen synthesis and storage is required to cope with different metabolic demands in insects. In *Drosophila,* adaptive mutations in the *Pgm1* locus show latitudinal clinal variation correlating with increased glycogen storage in temperate regions [67, 68]. Glycogen storage increases after blood meal in *R. prolixus*, is required for oogenesis and is completely depleted after prolonged starvation periods [69–72] (Fig. 6). Based on these, our observations could represent molecular adaptation in the *Pgm1* locus in reduviids, associated with an optimization in glycogen metabolic flux to cope with prolonged starvation and to achieve reproductive success.

The PPP, an early branch from glycolysis, is a major source of NADPH (oxidative phase) required for fatty acid synthesis and reactive oxygen species (ROS) scavenging via reduced glutathione. In its non-oxidative phase, it generates precursors for the synthesis of ribonucleotides. In *D. melanogaster,* an enhanced activity of the PPP oxidative phase induced by glucose 6-phosphate dehydrogenase (*G6pd*) overexpression, increases the levels of NADPH and extends the fly lifespan [73]. Among the *Triatoma* PPP enzymes, *Pgm2* and *Tald* belonged to the most divergent group, both mediate reactions in the non-oxidative phase. Ribose-5-phosphate isomerase (*Rpi*) generates the substrate for *Pgm2*, which in turn promotes ribonucleotide synthesis (Fig. 6). *Rpi* inhibition extends *Drosophila* life span [74]. *Tald* deficiency induces a starvation-like, mitochondrial stress, autophagy and extended life span in *Caenorhabditis elegans* [75]. This opens the possibility that the non-oxidative PPP may be implicated in contributing to the extended life span of reduviids.

The only glycolytic enzyme found in the most divergent group, *Pyk* mediates the last step of glycolysis, catalyzing the bidirectional inter-conversion between phosphoenolpyruvate and pyruvate (Fig. 6). Fructose 1, 6-bisphosphate is an allosteric activator of *Pyk*. Thus, gluconeogenesis and high *Fbp* activity suppresses *Pyk* [76]. The possibility that genetic variations in *Fbp* and *Pyk* may be functionally related deserves further study.

A clear overrepresentation of components of the respiratory chain complexes in the most divergent group was identified (Additional file 7); however, no evidence for positive selection was found using codon-based Z-tests, which are highly specific, although less sensitive for detecting selection. We reasoned that OxPhos proteins may be evolving under complex mechanisms of selection, and that signals for positive selection could be masked by an overwhelming number of sites under negative selection. Thus, we performed the position-based selection test with HyPhy. Although we did not find statistical significance in a per codon basis, a statistically significant bias towards positive dN-dS values was documented in the mitochondrial genome-encoded core subunits, and the β subcomplex subunits of NADH dehydrogenase complex (I); Cox7A subunit of the cytochrome oxidase C complex (IV) and the F_O_ - ATP synthase complex (V) subunits (Fig. 7), indicating increased likelihood of positive selection. Statistical significance in this method is highly dependent on the number of sequences included in the comparison (only four in this case), which could explain the lack of statistical significance in codons with positive dN-dS).

The NDUFA11 (α subcomplex, NADH dehydrogenase), NDUFB4, NDUFB8, NDUFB9 (β subcomplex of NADH dehydrogenase) and Cox7A2 mammalian homologs are implicated in the formation of respiratory chain “supercomplexes”, which may be required for adequate mitochondrial function [77]. Moreover, reactive oxygen species participate in ageing process in many metazoan models. The production of ROS by reverse electron transport, mediated by complex I or RNAi-mediated inhibition of complex I, III, IV and V, is associated with extended life span in *Drosophila* [78, 79]. Also, several complex I, IV and V mitochondria-encoded genes evolve under positive selection in flying insects, including hemipterans, but not in flightless insect orders [80].

We have based our interpretation of position-based selection test *p* values in the context of biological plausibility (i.e., examples of selection in OxPhos genes in other insects), a coherent effect in protein divergence in certain functionally related genes, and an equally coherent biased distribution of dN-dS of certain, but not all OxPhos sub-complexes (i.e., Fo but not F1 ATP synthase) to suggest, although not prove, the likelihood of selection in complexes I, IV and V. These observations suggest that triatomines underwent molecular adaptations in oxidative phosphorylation which may have resulted in optimization of energy metabolism and ROS generation balance. This optimization may allow these insects, on the one hand, to cope with prolonged starvation periods and on the other, the production the high amounts of energy demanded during hematophagy [81]. Their enhanced capacity to scavenge ROS by NADPH could contribute to their extended lifespan and might also be linked to their vectorial capacity, as redox status has been suggested to trigger changes in the life cycle of *Trypanosoma cruzi* in the vector [82].

## Conclusions

Overall, our results documented a set of shared gene expansions in triatomines possibly related to biological adaptations to their lifestyles and diverse ecological niches. Protease inhibitor and calycin-coding gene expansions points at rapidly evolving processes of protease regulation and hematophagy, which require further research for a better understanding of their role in vectorial capacity. Higher evolutionary rates in critical enzymes that exert metabolic flux control towards glycogen synthesis and the PPP, in oxidative phosphorylation complexes, and the expansion of the thioredoxin fold-containing proteins could represent genetic adaptations favoring hallmarks of triatomine life styles. Particularly, glycogen storage to cope with prolonged starvation and reproductive success, antioxidant mechanisms to cope with the oxidative stress favoring longevity, and optimization of aerobic metabolism to meet the energetic demands of hematophagy. Overall, this work represents a source of novel hypotheses related to triatomine biology to guide experimental testing.

## Methods

### Insect rearing

Colonies of *T. dimidiata* (colony 0252 from Tegucigalpa, Honduras), *T. infestans* (colony X32 from Santiago del Estero, Argentina) and *T. pallidipennis* (colony 0230 from Mexico) established in Centro Nacional de Chagas, Córdoba, Argentina were reared in the Centro Regional de Estudios Genómicos (CREG), Universidad Nacional de La Plata (UNLP) and the Centro de Bioinvestigaciones, Universidad Nacional del Noroeste de Buenos Aires (UNNOBA), at 28 °C and a partial humidity of 70% with a 12 h light/dark schedule. Insects were regularly fed using an artificial feeder and chicken blood. Insect handling was performed in accordance to the World Health Organization protocol [5].

#### Transcriptome preparation and sequencing

A mixture of RNA derived from different life-cycle stages of *T. infestans, T. dimidiata* and *T. pallidipennis* was used to generate a normalized cDNA library for each species as described [83]. Libraries were barcoded and subjected to the shotgun sequencing protocol using the GS FLX+ (454-Roche). Raw sequence datasets are available at the NCBI-SRA: *T. infestans* (SRX2600754), *T. dimidiata* (SRX2600753) and *T. pallidipennis* (SRX2600752).

#### Data filtering, trimming and assembly

Raw reads were analyzed with PRINSEQ [84] and filtered according to length, sequence complexity and quality. Each library was subjected to de novo assembly with the GS DeNovo assembler v.2.8 software in cDNA mode using the default parameters, and including the adaptor sequences for trimming. The assembled sequences dataset are available at the NCBI-TSA (GFMK00000000, GFMC00000000, GFMJ01000000). The non-assembled reads were mapped to the *R. prolixus* genome (Rhodnius-prolixus-CDC_SCAFFOLDS_RproC3.fa) using BLASTN and proteome (Rhodnius-prolixus-CDC_PEPTIDES_RproC3.2.fa) using BLASTX. Non-redundant mapped reads to either database were included as singletons in to the assembled dataset (*full_dataset*).

To mitigate the potential effects of redundancy in the *full_dataset* in the assessment of statistical representation of GO and InterPro annotations, two additional sequence databases for each species were built. A non-redundant database (*nr_dataset*) discards alternative isotigs belonging to the same isogroup or unigene, by keeping the largest isotig (transcript) per isogroup. The third database includes only non-redundant isotigs (*isotig_dataset*). An additional transcriptome database was created for each Triatoma species, by mapping raw reads to the R. prolixus predicted transcript dataset (Rhodnius-prolixus-CDC_TRANSCRIPTS_RproC3.2.fa) with the GS (Newbler) Mapper v.2.8. This dataset (*RproC3.2_mapped*) was used only for the identification of metabolic pathways enzyme-coding transcripts (Fig. 6. Additional file 8). To estimate the proportion of reads coded by the mitochondrial genome and to identify mitochondrial-encoded genes, the GS Mapper v 2.8 was used to map raw reads of each species to the *T. dimidiata* mitochondrial genome (Genbank accession: NC_002609.1) [22]. The same was done for the *Triatoma virus* genome (Genbank accession: NC_003783.1) (Table 2). All datasets used in this work are available in http://201.131.57.23:8080/data/triatoma.

#### Transcriptome completeness analysis

The assembled dataset for each species was used to identify the proportion of the core eukaryotic genome coverage, as described [83]. We used HMM profiles for 458 core eukaryotic proteins as provided by the CEGMA dataset [23] and HMMER3 searches with the *hmmscan* command and the -T 40 and --domT 40 filters, as described in [62]. Following the same approach, a Benchmarking Universal Single-Copy Orthologs (BUSCO) sets for arthropod [24] was used to assess transcriptome dataset completeness.

To estimate the proportion of each transcriptome database that is homologous to the *R. prolixus* predicted proteome [14], we used NCBI-BLASTX (−e 1.0E-05). Putative 1:1 orthologue identification between *R. prolixus* and the three - *Triatoma* transcriptomes was done using the BLAST best reciprocal hit strategy as described [62].

#### Insect proteome comparisons

The *full_dataset* transcriptome assemblies were used to find homolog proteins using BLASTX (cut-off –e 1.0E-05) in the following arthropod proteome databases (*Ixodes scapularis*, *Pediculus humanus*, *Lutzomya longipalpis*, *R. prolixus*, *Phlebotomus papatasi*, *Culex quinquefaciatus*, *Anopheles gambiae*, *Aedes aegypti*, and *Glossina morsitans*) downloaded from VectorBase [85], as well as *D. melanogaster* [86] and *Ac. pisum* [61].

#### Gene ontology and Interpro annotation

To classify transcripts according to their putative biological process, molecular function and structural relationships (protein conserved domains), these were analyzed according to Gene Ontology (GO) “terms” [87] and InterPro [25] annotations using the software package BLAST2Go Pro [26]. For the initial step, BLASTX against the NCBI-nr database, with a cut-off *e value* of 1.0E-6, was used. Once annotated, Gene Ontology term enrichment using as input the *isotig_dataset* was performed by means of Fisher’s exact test followed by a *P* value adjustment to correct for multiple testing with the Benjamini-Hochberg method (cut-off FDR < 0.05).

#### Identification of putative gene family expansions

The absolute count of InterPro annotations in the non-redundant *isotig_dataset* was used to estimate enrichment, using the *R. prolixus* InterPro annotations as reference. To avoid spurious enrichments, repeated InterPro ID’s in the same transcripts/gene were counted only once. A Fisher Exact Test, a 2 X 2 contingency table was built between the number of annotated and the non-annotated transcripts for a given IPR entry for each Triatoma species (*test*), as well as for *R. prolixus* (*reference*). The H_0_ is that there is no difference in the probability distribution between species and belonging to a given IPR entry. Given that transcript underrepresentation in transcriptome analysis in meaningless due the lack of certainty of completeness, emphasis was put in enrichment of IPR terms in Triatoma (Ha) by using a one-sided Fisher’s test with the R function fisher.test (c, alternative = “greater”), which indicates an enrichment in the *test* dataset. The function fisher.test (c, alternative = “less”) was also used to complement the enrichment analysis within Triatoma species, which indicates depletion in the *reference* dataset. P value adjustment with the Benjamini-Hochberg method was performed to correct for multiple testing using the R function p.adjust(p, method = “bh”, n = length(p)). A False Discovery Rate (FDR) < 0.05 was considered as a significant enrichment [88]. Individual protein family analysis was complemented with local InterProScan analysis [25] or HMMER3 searches using specific PFAM profiles [89].

#### Transposable element analysis

Analysis of expressed transposable elements (TEs) was performed with local NCBI-BLASTN, with a cut-off *e value* of 1.0E-6 against the RepBase database, Version 21.08 [35]. Matches with alignment lengths lesser than 100 bps were discarded. Analysis was only focused in DNA transposons (Class II), LTR and non-LTR retrotransposons.

#### Cluster analysis of the calycin/lipocalin family

To analyze the global phylogenetic relationships of our calycin/lipocalin ortholog predictions, we performed a clustering analysis with CLANS software [32]. CLANS is a useful tool to generate 3D clustering many sequences based on their sequence similarity. It uses a variant of the Fruchterman and Reingold graph layout algorithm to generate graphs providing graphical representation of pairwise sequence similarities. Sequences are represented by vertices in the graph, BLAST/PSIBLAST high scoring segment pairs (HSPs) are shown as edges connecting vertices and provide attractive forces proportional to the negative algorithm of the HSP’s P -value. In this way, similar sequences reproducibly cluster after a few iterations. The parameters used for the analysis were as follows: PSI-BLAST as sequence-similarity-based approach, BLOSUM62 matrix, *P*-value cutoff of 1e − 37, repuls = 2 and attract = 2.

#### Estimation of divergence in ortholog pairs according to GO

The sequence identity (%) for each 1:1 *R. prolixus - Triatoma* ortholog pair identified by BLAST – best reciprocal hit strategy (*isotig_dataset*) was grouped according to Gene Ontology biological process, molecular function and cellular component categories (Level 3). Median % identity for each category was used to calculate Z values (number of standard deviations from the % identity mean) by subtracting the overall median % identity (all GO annotated orthologs). Statistical significance (*p* < 0.05) was determined with Kruskal-Wallis one-way ANOVA test with Dunn’s correction for multiple comparisons. Z values were hierarchically clustered using Cluster 3.0 [90] according to species and graphically represented as heatmaps using Java TreeView 1.1.6r4 (http://jtreeview.sourceforge.net).

To study protein sequence divergence according to more specific GO terms, the whole set of GO 1:1 orthologs (*isotig-dataset*) was subdivided into equal terciles according to % identity (BLASTx) (i.e. more, average and less-conserved terciles or groups) (Additional file 6). To identify more specific GO categories that were enriched in the less conserved group, a Fisher Exact Test was performed using the GO annotations in the most conserved group as reference using BLAST2Go Pro [26]. *P* values were adjusted for multiple testing using the Benjamini-Hochberg method. A FDR < 0.05 was considered significant.

#### Selection analysis of the respiratory chain complexes

To test selection in coding genes of the oxidative phosphorylation complexes, orthologs nucleotide sequences of the four species were codon-aligned. All ambiguous positions and gaps were removed for each sequence pair. Two approaches were used. The first was a codon-based Z-test of positive selection in which the variance of the difference of the numbers of synonymous (dS) and nonsynonymous (dN) substitutions per site was computed using the Nei-Gojobori method [91]. In the second approach, nucleotide sequences were subjected to a codon-by-codon Maximum Likelihood analysis with the HyPhy package [42] using the Muse-Gaut model [92] of codon substitution and Felsenstein model [93] of nucleotide substitution. The median of normalized dN-dS for each variant position was calculated and plotted according to increasing median value. Statistical significance was evaluated with a Kruskal-Wallis test with Dunn’s multiple comparisons correction between the sequence with the lowest-median dN-dS and the remaining sequences. Evolutionary analyses were conducted in MEGA7 [94].

## Additional files

Additional file 1: InterProScan analysis of Pacifastin domain containing proteins in Reduviids (IPR008037). Data for Fig. 2. (XLS 40 kb)

Additional file 2: Analysis of the calycin family in *Triatoma*. Data for Fig. 3. (XLS 58 kb)

Additional file 3: Transcriptionally active transposable elements in *Triatoma*. BLASTn analysis search in the RepBase database. Data for Fig. 4. (XLS 64 kb)

Additional file 4: Transcripts coding for odorant receptors and odorant binding proteins in *Triatoma*. (XLS 78 kb)

Additional file 5: Protein sequence divergence in putative 1:1 orthologs according to Gene Ontology terms (Level 3). Isotig dataset. Data for Fig. 5. (XLS 54 kb)

Additional file 6: Sequence identity distribution for *R. prolixus-Triatoma* 1:1 putative orthologs divided into equal terciles or groups. (PDF 450 kb)

Additional file 7: Gene ontology term enrichment in divergent vs. conserved group putative 1:1 orthologs (isotig dataset). (XLS 43 kb)

Additional file 8: Identification of Glycolysis, gluconeogenesis and pentose-phosphate putative orthologs and classification according to protein sequence conservation (RproC3.2_mapped dataset. Data for Fig. 6. (XLS 62 kb)

Additional file 9: Oxidative phosphorylation. Identification of respiratory complex subunit putative orthologs and classification according to protein sequence conservation (*nr-dataset* and *mitochondrial_mapped* datasets. Data for Fig. 7) (XLS 69 kb)

## Abbreviations

ERV: Endogenous retrovirus
FABP: Fatty acid binding protein
FDR: False discovery rate
HT: Horizontal transfer
LTR: Long terminal repeat
non-LTR: Non-long terminal repeat
OBP: Odorant binding protein
OxPhos: Oxidative phosphorylation
PPP: Pentose phosphate pathway
ROS: Reactive oxygen species
*T. cruzi*: *Trypanosoma cruzi*
TCA: Tricarboxylic acid
TE: Transposable element
VWFC: von Willebrand factor, type C domain
VWFD: von Willebrand factor, type D domain

## References

[1] Lent HWP (1979). Revision of the Triatominae (Hemiptera, Reduviidae)**,** and their significance as vectors of Chagas’ disease, vol 163.

[2] Justi SA, Galvao C (2017). The evolutionary origin of diversity in Chagas disease vectors. Trends Parasitol.

[3] Hwang WS, Weirauch C (2012). Evolutionary history of assassin bugs (insecta: hemiptera: Reduviidae): insights from divergence dating and ancestral state reconstruction. PLoS One.

[4] Gaunt M, Miles M (2000). The Ecotopes and evolution of Triatomine bugs (Triatominae) and their associated trypanosomes. Mem Inst Oswaldo Cruz.

[5] WHO (2002). Control of Chagas disease. World Health Organization Tech Rep Ser.

[6] Fitzpatrick S, Feliciangeli MD, Sanchez-Martin MJ, Monteiro FA, Miles MA (2008). Molecular genetics reveal that silvatic Rhodnius prolixus do colonise rural houses. PLoS Negl Trop Dis.

[7] Rassi A, Rassi A, Marin-Neto JA (2010). Chagas disease. Lancet.

[8] Zeledon R, Guardia VM, Zuniga A, Swartzwelder JC (1970). Biology and ethology of Triatoma dimidiata (Latreille, 1811). II. Life span of adults and fecundity and fertility of females. J Med Entomol.

[9] Zeledon R, Guardia VM, Zuniga A, Swartzwelder JC (1970). Biology and ethology of Triatoma dimidiata (Latreille, 1811). I. Life cycle, amount of blood ingested, resistance of starvation, and size of adults. J Med Entomol.

[10] Monroy MC, Bustamante DM, Rodas AG, Enriquez ME, Rosales RG (2003). Habitats, dispersion and invasion of sylvatic Triatoma dimidiata (Hemiptera: Reduviidae: Triatominae) in Peten, Guatemala. J Med Entomol.

[11] Ramsey JM, Peterson AT, Carmona-Castro O, Moo-Llanes DA, Nakazawa Y, Butrick M, Tun-Ku E, la Cruz-Felix K, Ibarra-Cerdena CN (2015). Atlas of Mexican Triatominae (Reduviidae: Hemiptera) and vector transmission of Chagas disease. Mem Inst Oswaldo Cruz.

[12] Ramsey JM, Ordonez R, Cruz-Celis A, Alvear AL, Chavez V, Lopez R, Pintor JR, Gama F, Carrillo S (2000). Distribution of domestic triatominae and stratification of Chagas disease transmission in Oaxaca, Mexico. Med Vet Entomol.

[13] Gurtler RE (2009). Sustainability of vector control strategies in the gran Chaco region: current challenges and possible approaches. Mem Inst Oswaldo Cruz.

[14] Mesquita RD, Vionette-Amaral RJ, Lowenberger C, Rivera-Pomar R, Monteiro FA, Minx P, Spieth J, Carvalho AB, Panzera F, Lawson D (2015). Genome of Rhodnius prolixus, an insect vector of Chagas disease, reveals unique adaptations to hematophagy and parasite infection. Proc Natl Acad Sci U S A.

[15] Ribeiro JMC, Assumpcao TCF, Pham VM, Francischetti IMB, Reisenman CE (2012). An insight into the Sialotranscriptome of Triatoma rubida (Hemiptera: Heteroptera). J Med Entomol.

[16] Assumpcao TC, Francischetti IM, Andersen JF, Schwarz A, Santana JM, Ribeiro JM (2008). An insight into the sialome of the blood-sucking bug Triatoma infestans, a vector of Chagas' disease. Insect Biochem Mol Biol.

[17] Assumpcao TC, Eaton DP, Pham VM, Francischetti IM, Aoki V, Hans-Filho G, Rivitti EA, Valenzuela JG, Diaz LA, Ribeiro JM (2012). An insight into the sialotranscriptome of Triatoma matogrossensis, a kissing bug associated with fogo selvagem in South America. American J Trop Med Hygiene.

[18] Ribeiro JM, Schwarz A, Francischetti IM (2015). A deep insight into the Sialotranscriptome of the Chagas disease vector, Panstrongylus megistus (Hemiptera: Heteroptera). J Med Entomol.

[19] Traverso L, Lavore A, Sierra I, Palacio V, Martinez-Barnetche J, Latorre-Estivalis JM, Mougabure-Cueto G, Francini F, Lorenzo MG, Rodriguez MH (2017). Comparative and functional triatomine genomics reveals reductions and expansions in insecticide resistance-related gene families. PLoS Negl Trop Dis.

[20] Hernandez-Vargas MJ, Santibanez-Lopez CE, Corzo G (2016). An insight into the Triabin protein family of American hematophagous Reduviids: functional, structural and phylogenetic analysis. Toxins.

[21] Marchant A, Mougel F, Almeida C, Jacquin-Joly E, Costa J, Harry M (2015). De novo transcriptome assembly for a non-model species, the blood-sucking bug Triatoma brasiliensis, a vector of Chagas disease. Genetica.

[22] Dotson EM, Beard CB (2001). Sequence and organization of the mitochondrial genome of the Chagas disease vector, Triatoma dimidiata. Insect Mol Biol.

[23] Parra G, Bradnam K, Korf I (2007). CEGMA: a pipeline to accurately annotate core genes in eukaryotic genomes. Bioinformatics.

[24] Simao FA, Waterhouse RM, Ioannidis P, Kriventseva EV, Zdobnov EM (2015). BUSCO: assessing genome assembly and annotation completeness with single-copy orthologs. Bioinformatics.

[25] Jones P, Binns D, Chang HY, Fraser M, Li W, McAnulla C, McWilliam H, Maslen J, Mitchell A, Nuka G (2014). InterProScan 5: genome-scale protein function classification. Bioinformatics.

[26] Gotz S, Garcia-Gomez JM, Terol J, Williams TD, Nagaraj SH, Nueda MJ, Robles M, Talon M, Dopazo J, Conesa A (2008). High-throughput functional annotation and data mining with the Blast2GO suite. Nucleic Acids Res.

[27] Breugelmans B, Simonet G, van Hoef V, Van Soest S, Vanden Broeck J (2009). Pacifastin-related peptides: structural and functional characteristics of a family of serine peptidase inhibitors. Peptides.

[28] Wallin H, Bjarnadottir M, Vogel LK, Wasselius J, Ekstrom U, Abrahamson M (2010). Cystatins--extra- and intracellular cysteine protease inhibitors: high-level secretion and uptake of cystatin C in human neuroblastoma cells. Biochimie.

[29] Turk V, Stoka V, Turk D (2008). Cystatins: biochemical and structural properties, and medical relevance. Front Biosci.

[30] Neafsey DE, Waterhouse RM, Abai MR, Aganezov SS, Alekseyev MA, Allen JE, Amon J, Arca B, Arensburger P, Artemov G (2015). Mosquito genomics. Highly evolvable malaria vectors: the genomes of 16 Anopheles mosquitoes. Science.

[31] Kato H, Jochim RC, Gomez EA, Sakoda R, Iwata H, Valenzuela JG, Hashiguchi Y (2010). A repertoire of the dominant transcripts from the salivary glands of the blood-sucking bug, Triatoma dimidiata, a vector of Chagas disease. Infect Genet Evol.

[32] Frickey T, Lupas A (2004). CLANS: a java application for visualizing protein families based on pairwise similarity. Bioinformatics.

[33] Montfort WR, Weichsel A, Andersen JF (2000). Nitrophorins and related antihemostatic lipocalins from Rhodnius prolixus and other blood-sucking arthropods. Biochim Biophys Acta.

[34] Ribeiro JM, Genta FA, Sorgine MH, Logullo R, Mesquita RD, Paiva-Silva GO, Majerowicz D, Medeiros M, Koerich L, Terra WR (2014). An insight into the transcriptome of the digestive tract of the bloodsucking bug, Rhodnius prolixus. PLoS Negl Trop Dis.

[35] Bao W, Kojima KK, Kohany O (2015). Repbase update, a database of repetitive elements in eukaryotic genomes. Mob DNA.

[36] Potter CJ (2014). Stop the biting: targeting a mosquito's sense of smell. Cell.

[37] Rebers JE, Willis JH (2001). A conserved domain in arthropod cuticular proteins binds chitin. Insect Biochem Mol Biol.

[38] Suetake T, Tsuda S, Kawabata S, Miura K, Iwanaga S, Hikichi K, Nitta K, Kawano K (2000). Chitin-binding proteins in invertebrates and plants comprise a common chitin-binding structural motif. J Biol Chem.

[39] Alvarenga ES, Mansur JF, Justi SA, Figueira-Mansur J, Dos Santos VM, Lopez SG, Masuda H, Lara FA, Melo AC, Moreira MF (2016). Chitin is a component of the Rhodnius prolixus midgut. Insect Biochem Mol Biol.

[40] Soares JB, Gaviraghi A, Oliveira MF (2015). Mitochondrial physiology in the major arbovirus vector Aedes aegypti: substrate preferences and sexual differences define respiratory capacity and superoxide production. PLoS One.

[41] Brand MD (2016). Mitochondrial generation of superoxide and hydrogen peroxide as the source of mitochondrial redox signaling. Free Radic Biol Med.

[42] Kosakovsky Pond SL, Frost SD (2005). Not so different after all: a comparison of methods for detecting amino acid sites under selection. Mol Biol Evol.

[43] Waterhouse RM (2015). A maturing understanding of the composition of the insect gene repertoire. Current Opin Insect Sci.

[44] Feyereisen R (2011). Arthropod CYPomes illustrate the tempo and mode in P450 evolution. Biochim Biophys Acta.

[45] Liang Z, Sottrup-Jensen L, Aspan A, Hall M, Soderhall K (1997). Pacifastin, a novel 155-kDa heterodimeric proteinase inhibitor containing a unique transferrin chain. Proc Natl Acad Sci U S A.

[46] Breugelmans B, Simonet G, van Hoef V, Van Soest S, Broeck JV (2009). Identification, distribution and molecular evolution of the pacifastin gene family in Metazoa. BMC Evol Biol.

[47] Schwarz A, Medrano-Mercado N, Schaub GA, Struchiner CJ, Bargues MD, Levy MZ, Ribeiro JM (2014). An updated insight into the Sialotranscriptome of Triatoma infestans: developmental stage and geographic variations. PLoS Negl Trop Dis.

[48] de Marco R, Lovato DV, Torquato RJ, Clara RO, Buarque DS, Tanaka AS (2010). The first pacifastin elastase inhibitor characterized from a blood sucking animal. Peptides.

[49] Kotsyfakis M, Karim S, Andersen JF, Mather TN, Ribeiro JM (2007). Selective cysteine protease inhibition contributes to blood-feeding success of the tick Ixodes scapularis. J Biol Chem.

[50] Kotsyfakis M, Sa-Nunes A, Francischetti IM, Mather TN, Andersen JF, Ribeiro JM (2006). Antiinflammatory and immunosuppressive activity of sialostatin L, a salivary cystatin from the tick Ixodes scapularis. J Biol Chem.

[51] Assumpcao TC, Charneau S, Santiago PB, Francischetti IM, Meng Z, Araujo CN, Pham VM, Queiroz RM, de Castro CN, Ricart CA (2011). Insight into the salivary transcriptome and proteome of Dipetalogaster maxima. J Proteome Res.

[52] Buarque DS, Spindola LM, Martins RM, Braz GR, Tanaka AS (2011). Tigutcystatin, a cysteine protease inhibitor from Triatoma infestans midgut expressed in response to Trypanosoma cruzi. Biochem Biophys Res Commun.

[53] Flower DR, North AC, Sansom CE (2000). The lipocalin protein family: structural and sequence overview. Biochim Biophys Acta.

[54] Gerstner JR, Vanderheyden WM, Shaw PJ, Landry CF, Yin JC (2011). Fatty-acid binding proteins modulate sleep and enhance long-term memory consolidation in Drosophila. PLoS One.

[55] Panzera F, Ferrandis I, Ramsey J, Salazar-Schettino PM, Cabrera M, Monroy C, Bargues MD, Mas-Coma S, O'Connor JE, Angulo VM (2007). Genome size determination in chagas disease transmitting bugs (hemiptera-triatominae) by flow cytometry. Am J Tropical Med Hyg.

[56] Habibi L, Pedram M, AmirPhirozy A, Bonyadi K (2015). Mobile DNA elements: the seeds of organic complexity on earth. DNA Cell Biol.

[57] Gilbert C, Schaack S, Pace JK, Brindley PJ, Feschotte C (2010). A role for host-parasite interactions in the horizontal transfer of transposons across phyla. Nature.

[58] Sormacheva I, Smyshlyaev G, Mayorov V, Blinov A, Novikov A, Novikova O (2012). Vertical evolution and horizontal transfer of CR1 non-LTR retrotransposons and Tc1/mariner DNA transposons in Lepidoptera species. Mol Biol Evol.

[59] Novikova O, Sliwinska E, Fet V, Settele J, Blinov A, Woyciechowski M (2007). CR1 clade of non-LTR retrotransposons from Maculinea butterflies (Lepidoptera: Lycaenidae): evidence for recent horizontal transmission. BMC Evol Biol.

[60] Biedler JK, Chen X, Tu Z (2015). Horizontal transmission of an R4 clade non-long terminal repeat retrotransposon between the divergent Aedes and Anopheles mosquito genera. Insect Mol Biol.

[61] International Aphid Genomics C (2010). Genome sequence of the pea aphid Acyrthosiphon pisum. PLoS Biol.

[62] Martinez-Barnetche J, Gomez-Barreto RE, Ovilla-Munoz M, Tellez-Sosa J, Garcia Lopez DE, Dinglasan RR, Ubaida Mohien C, MacCallum RM, Redmond SN, Gibbons JG (2012). Transcriptome of the adult female malaria mosquito vector Anopheles albimanus. BMC Genomics.

[63] Eanes WF (2011). Molecular population genetics and selection in the glycolytic pathway. J Exp Biol.

[64] Zera AJ (2011). Microevolution of intermediary metabolism: evolutionary genetics meets metabolic biochemistry. J Exp Biol.

[65] Leite A, Neto JA, Leyton JF, Crivellaro O, el-Dorry HA (1988). Phosphofructokinase from bumblebee flight muscle. Molecular and catalytic properties and role of the enzyme in regulation of the fructose 6-phosphate/fructose 1,6-bisphosphate cycle. J Biol Chem.

[66] Staples JF, Koen EL, Laverty TM (2004). Futile cycle' enzymes in the flight muscles of north American bumblebees. J Exp Biol.

[67] Verrelli BC, Eanes WF (2001). The functional impact of Pgm amino acid polymorphism on glycogen content in Drosophila melanogaster. Genetics.

[68] Verrelli BC, Eanes WF (2001). Clinal variation for amino acid polymorphisms at the Pgm locus in Drosophila melanogaster. Genetics.

[69] Wigglesworth VB (1967). Cytological changes in the fat body of Rhodnius during starvation, feeding and oxygen want. J Cell Sci.

[70] Santos R, Mariano AC, Rosas-Oliveira R, Pascarelli B, Machado EA, Meyer-Fernandes JR, Gondim KC (2008). Carbohydrate accumulation and utilization by oocytes of Rhodnius prolixus. Arch Insect Biochem Physiol.

[71] Mariano AC, Santos R, Gonzalez MS, Feder D, Machado EA, Pascarelli B, Gondim KC, Meyer-Fernandes JR (2009). Synthesis and mobilization of glycogen and trehalose in adult male Rhodnius prolixus. Arch Insect Biochem Physiol.

[72] Mury FB, Lugon MD, RN DAF, Silva JR, Berni M, Araujo HM, Fontenele MR, Abreu LA, Dansa M, Braz G (2016). Glycogen synthase Kinase-3 is involved in glycogen metabolism control and embryogenesis of Rhodnius prolixus. Parasitology.

[73] Legan SK, Rebrin I, Mockett RJ, Radyuk SN, Klichko VI, Sohal RS, Orr WC (2008). Overexpression of glucose-6-phosphate dehydrogenase extends the life span of Drosophila melanogaster. J Biol Chem.

[74] Wang CT, Chen YC, Wang YY, Huang MH, Yen TL, Li H, Liang CJ, Sang TK, Ciou SC, Yuh CH (2012). Reduced neuronal expression of ribose-5-phosphate isomerase enhances tolerance to oxidative stress, extends lifespan, and attenuates polyglutamine toxicity in Drosophila. Aging Cell.

[75] Bennett CF, Kwon JJ, Chen C, Russell J, Acosta K, Burnaevskiy N, Crane MM, Bitto A, Vander Wende H, Simko M (2017). Transaldolase inhibition impairs mitochondrial respiration and induces a starvation-like longevity response in Caenorhabditis elegans. PLoS Genet.

[76] Dayton TL, Jacks T, Vander Heiden MG (2016). PKM2, cancer metabolism, and the road ahead. EMBO Rep.

[77] Milenkovic D, Blaza JN, Larsson NG, Hirst J (2017). The enigma of the respiratory chain Supercomplex. Cell Metab.

[78] Scialo F, Sriram A, Fernandez-Ayala D, Gubina N, Lohmus M, Nelson G, Logan A, Cooper HM, Navas P, Enriquez JA (2016). Mitochondrial ROS produced via reverse Electron transport extend animal lifespan. Cell Metab.

[79] Copeland JM, Cho J, Lo T, Hur JH, Bahadorani S, Arabyan T, Rabie J, Soh J, Walker DW (2009). Extension of Drosophila life span by RNAi of the mitochondrial respiratory chain. Current Biol.

[80] Yang Y, Xu S, Xu J, Guo Y, Yang G (2014). Adaptive evolution of mitochondrial energy metabolism genes associated with increased energy demand in flying insects. PLoS One.

[81] Leis M, Pereira MH, Casas J, Menu F, Lazzari CR (2016). Haematophagy is costly: respiratory patterns and metabolism during feeding in Rhodnius prolixus. J Exp Biol.

[82] Nogueira NP, Saraiva FM, Sultano PE, Cunha PR, Laranja GA, Justo GA, Sabino KC, Coelho MG, Rossini A, Atella GC (2015). Proliferation and differentiation of Trypanosoma cruzi inside its vector have a new trigger: redox status. PLoS One.

[83] Zumaya-Estrada FA, Martinez-Barnetche J, Lavore A, Rivera-Pomar R, Rodriguez MH (2018). Comparative genomics analysis of triatomines reveals common first line and inducible immunity-related genes and the absence of Imd canonical components among hemimetabolous arthropods. Parasit Vectors.

[84] Schmieder R, Edwards R (2011). Quality control and preprocessing of metagenomic datasets. Bioinformatics.

[85] Giraldo-Calderon GI, Emrich SJ, MacCallum RM, Maslen G, Dialynas E, Topalis P, Ho N, Gesing S, VectorBase C, Madey G (2015). VectorBase: an updated bioinformatics resource for invertebrate vectors and other organisms related with human diseases. Nucleic Acids Res.

[86] Adams MD, Celniker SE, Holt RA, Evans CA, Gocayne JD, Amanatides PG, Scherer SE, Li PW, Hoskins RA, Galle RF (2000). The genome sequence of Drosophila melanogaster. Science.

[87] Gene Ontology C (2015). Gene ontology consortium: going forward. Nucleic Acids Res.

[88] Rivals I, Personnaz L, Taing L, Potier MC (2007). Enrichment or depletion of a GO category within a class of genes: which test?. Bioinformatics.

[89] Finn RD, Bateman A, Clements J, Coggill P, Eberhardt RY, Eddy SR, Heger A, Hetherington K, Holm L, Mistry J (2014). Pfam: the protein families database. Nucleic Acids Res.

[90] Eisen MB, Spellman PT, Brown PO, Botstein D (1998). Cluster analysis and display of genome-wide expression patterns. Proc Natl Acad Sci U S A.

[91] Nei M, Gojobori T (1986). Simple methods for estimating the numbers of synonymous and nonsynonymous nucleotide substitutions. Mol Biol Evol.

[92] Muse SV, Gaut BS (1994). A likelihood approach for comparing synonymous and nonsynonymous nucleotide substitution rates, with application to the chloroplast genome. Mol Biol Evol.

[93] Felsenstein J (1981). Evolutionary trees from DNA sequences: a maximum likelihood approach. J Mol Evol.

[94] Kumar S, Stecher G, Tamura K (2016). MEGA7: molecular evolutionary genetics analysis version 7.0 for bigger datasets. Mol Biol Evol.

