# Supplementary material for: Adaptations in energy metabolism and gene family expansions revealed by comparative transcriptomics of three Chagas disease triatomine vectors
Source: BMC Genomics. 2018 Apr 27;19:296. doi: 10.1186/s12864-018-4696-8 (PMC5921304; doi:10.1186/s12864-018-4696-8)
Supplement: Supplementary file 6 — Sequence identity distribution for R. prolixus-Triatoma 1:1 putative orthologs divided into equal terciles or groups. (PDF 450 kb) [file 12864_2018_4696_MOESM6_ESM.pdf]

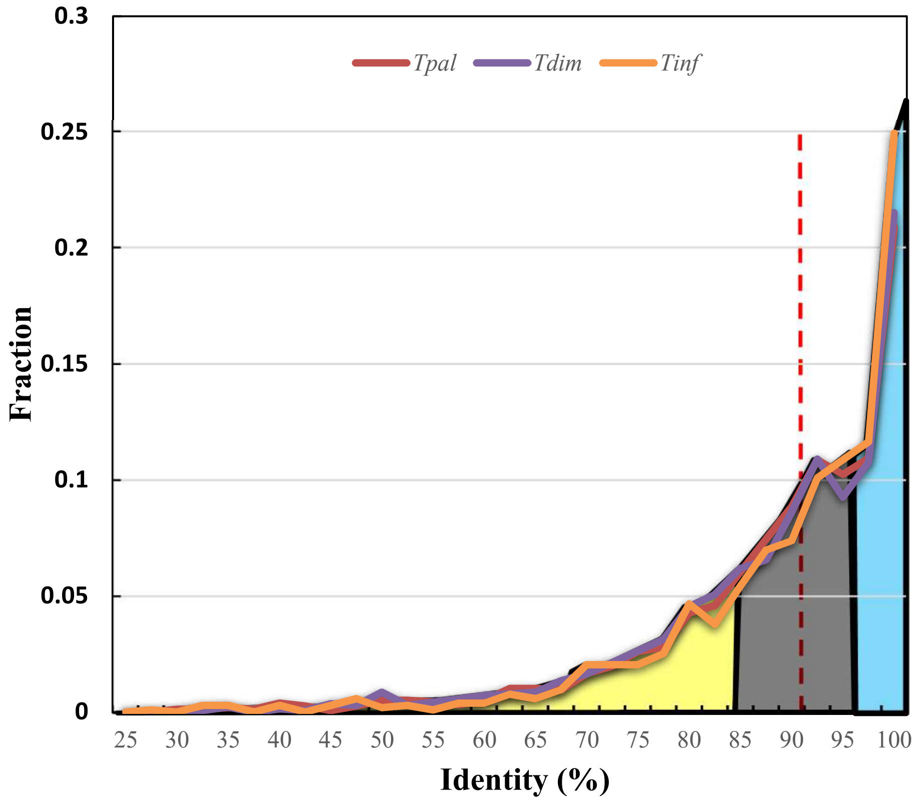

Additional file 6. One-to-one *Triatoma – R. prolixus* ortholog pairwise protein identity distribution. The proportion of orthologs for each species as a function of BLASTx pairwise identity using the BLAST - best reciprocal hit strategy. The whole ortholog dataset for each species was divided in equal terciles or groups. The most divergent tercile is shown in yellow. The most conserved tercile is shown in blue. The red dotted line represents the median used to calculate Z scores (**Figure. 5. Additional file 5**). Gene Ontology term enrichment in the most divergent tercile was determined by means of a Fisher's Exact test using the most conserved tercile as reference (FDR < 0.05). The respective GO terms over and underrepresentation in the most divergent tercile is detailed in **Additional file 7**.
